# Targeted Microbubbles for Drug, Gene, and Cell Delivery in Therapy and Immunotherapy

**DOI:** 10.3390/pharmaceutics15061625

**Published:** 2023-05-30

**Authors:** J. Angel Navarro-Becerra, Mark A. Borden

**Affiliations:** 1 Mechanical Engineering Department, University of Colorado Boulder, Boulder, CO 80309, USA; jose.navarro@colorado.edu; 2 Biomedical Engineering Program, University of Colorado Boulder, Boulder, CO 80309, USA

**Keywords:** targeted microbubbles, ultrasound imaging probes, ultrasound-targeted delivery, therapy, immunotherapy

## Abstract

Microbubbles are 1–10 μm diameter gas-filled acoustically-active particles, typically stabilized by a phospholipid monolayer shell. Microbubbles can be engineered through bioconjugation of a ligand, drug and/or cell. Since their inception a few decades ago, several targeted microbubble (tMB) formulations have been developed as ultrasound imaging probes and ultrasound-responsive carriers to promote the local delivery and uptake of a wide variety of drugs, genes, and cells in different therapeutic applications. The aim of this review is to summarize the state-of-the-art of current tMB formulations and their ultrasound-targeted delivery applications. We provide an overview of different carriers used to increase drug loading capacity and different targeting strategies that can be used to enhance local delivery, potentiate therapeutic efficacy, and minimize side effects. Additionally, future directions are proposed to improve the tMB performance in diagnostic and therapeutic applications.

## 1. Introduction

**Figure 1:**
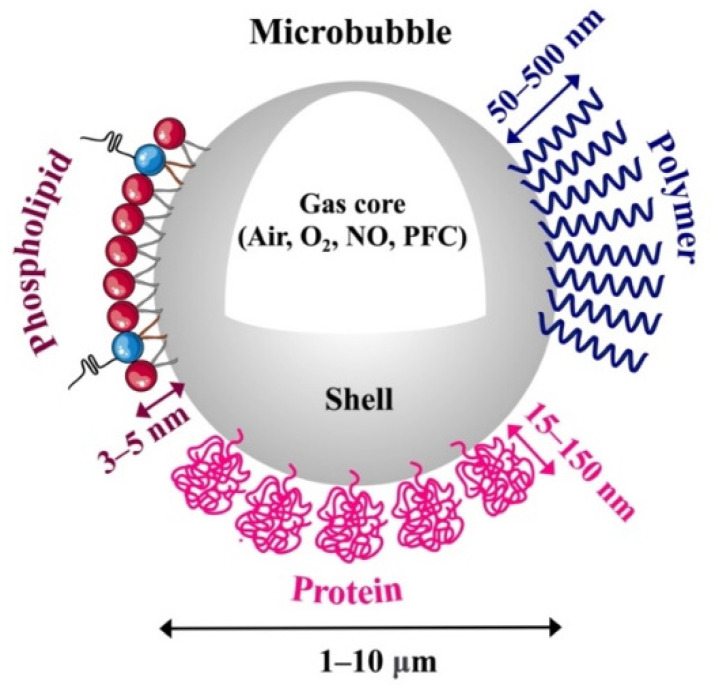
Illustration of a typical engineered microbubble used in biomedical applications. Microbubbles are particles filled with gas, such as air, oxygen (O_2_), nitric oxide (NO) or perfluorocarbon (PFC), with a diameter of 1–10 μm. They are stabilized by shells made of phospholipid (3–5 nm thick), protein (15–150 nm thick) or polymer (50–500 nm thick).

Microbubbles (MBs) are gas-filled particles of 1–10 μm in diameter suspended in an aqueous medium. The gas core is highly compressible, making the MB an ideal acoustically responsive agent for ultrasound. However, uncoated MBs are highly unstable owing to their Laplace pressure [1] and tendency to coalesce [2]. Therefore, shells comprising lipid, surfactant, protein, polymer or other materials have been developed to stabilize the MBs for in vitro and in vivo use (Figure 1) [3,4]. Current clinically approved MBs comprise sulfur hexafluoride, perfluorobutane or perfluoropropane gas, and a phospholipid or protein shell (Table 1) [4,5,6]. These commercial MBs have been approved as ultrasound contrast agents for different diagnostic purposes including echocardiography, radiology, and other diagnostic imaging purposes [5,6,7].

Following their clinical translation as ultrasound imaging contrast agents, MB formulations have been engineered as ultrasound-responsive carriers to promote and enhance the local delivery and uptake of a wide variety of drugs [8,9,10,11], genes [12,13,14], and cells [15,16,17,18,19,20] for various therapeutic applications. Many of these applications include the delivery of therapeutic agents to treat the brain [19,20,21,22,23], heart [15,24,25,26], and cancer [27,28,29]. For immunotherapy, MBs can facilitate the delivery of immune cells, cytokines, antigens, and antibodies to promote the activation and infiltration of immune cells from the level of single cells to tissues, organs, and even physiological systems [28,30,31,32]. In cancer, for example, the concept is to induce the modulation and modification of the tumor microenvironment with a subsequent enhanced adaptive immune-cell activation to destroy the primary tumor and its metastases.

**Figure 2:**
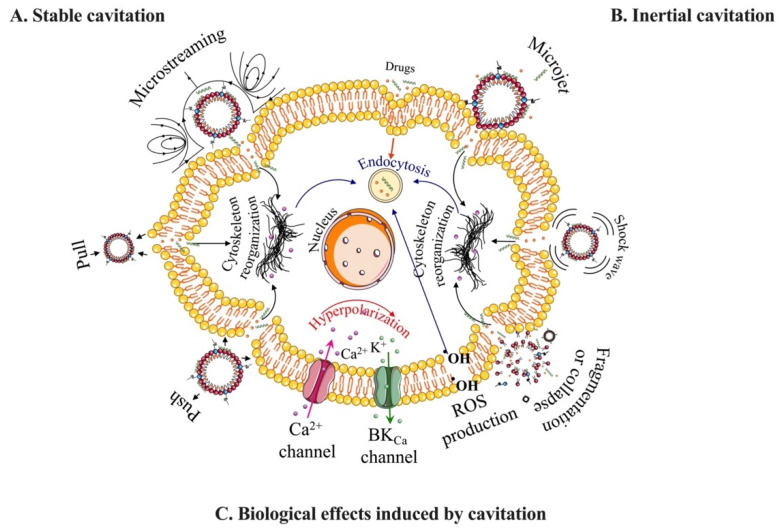
Schematic overview of the underlying physical and biological effects produced during microbubble cavitation on the cell membrane. (**A**,**B**) Mechanical effects associated with sonoporation, including stable and inertial cavitation of microbubbles. (**C**) Biological effects induced by microbubble cavitation, which enhance the internalization of drugs.

At the cellular level, increased uptake of therapeutic molecules into the cytosol has been attributed to sonoporation, i.e., the creation of a transient pore in the cell plasma membrane that increases permeability to molecules into the cytosol [33,34]. This effect is elicited by MB cavitation at the cell surface. MB cavitation refers to volumetric oscillations of the gas core and depends on the frequency, pressure, and waveform of the acoustic pulse. Fortuitously, MBs resonate at clinical ultrasound scanner frequencies, in the 1–10 MHz range. At low acoustic pressures, MBs exhibit stable cavitation, whereby repeated harmonic oscillations induce pushing-and-pulling effects and microstreaming that impart contact and shear forces against the cell membrane (Figure 2A) [35,36]. This mode of operation can be detected by passive acoustic cavitation and cavitation mapping as harmonics of the fundamental diving frequency [37]. At high acoustic pressures, MBs oscillate more violently, leading to rapid collapse and other inertial effects, such as microjets, shockwaves, and fragmentation (Figure 2B) [35,36]. This mode of operation can be detected by passive acoustic cavitation and cavitation mapping as a broadband response, although inertial cavitation can also enhance harmonics [37]. Stable and inertial cavitation can trigger various cellular effects, such as plasma membrane pore formation, cytoskeleton reorganization, transmembrane calcium (Ca^2+^) influx, potassium (K^+^) efflux (hyperpolarization), and reactive oxygen species production (Figure 2C). These biological effects lead to enhanced cellular delivery through mechanisms such as drug convection and diffusion through the pores (4–70 kDa) and endocytosis (70–500 kDa) [33,34,35,38].

**Table 1:** Current clinically approved MB ultrasound contrast agents.

Contrast Agent	Manufacturer	Indications	Shell	Gas	Concentration (MBs/mL)	Size (Diameter)	Half-Life (min)	Volume Dose (μL/kg) a	Mechanical Properties	Reference
**Optison**	GE Healthcare	LVO/EBD	Protein: HSA *ψ*_0_ = −9.5 to −25.3 mV	C_3_F_8_	5–8 × 10^8^	3.0–4.5 μm (max. 32 μm) 95% < 10 μm	0.5 ± 0.3	6	*f* = 2–4 MHz χ = 0.9 N/m	[39,40,41,42,43]
**Definity**	Lantheus	LVO/EBD, breast, liver, vascular.	Phospholipid: DPPC, DPPA, DPPE-mPEG5000 *ψ*_0_ = −1.1 to −4.2 mV	C_3_F_8_	1.2 × 10^10^	1.1–3.3 (max. 20 μm) 98% < 10 μm	2.0 ± 0.3	10	*f* = 2–6 MHz χ = 0.5–2.5 N/m	[40,42,43,44,45,46]
**SonoVue**	Bracco	LVO/EBD, breast, liver, vascular, urinary tract.	Phospholipid: DSPC, DPPG, PA *ψ*_0_ = −28.3 mV	SF_6_	1.5–2.5 × 10^8^	1.5–2.5 (max. 20 μm) 99% < 10 μm	1.04 ± 0.15	25	*f* = 1.5–2 MHz χ = 0.2–0.3 N/m	[40,44,47,48,49]
**Sonazoid**	GE Healthcare	Myocardial perfusion, liver, breast.	Phospholipid: H-EPS *ψ*_0_ = −76 to −82 mV	C_4_F_10_	1.2 × 10^9^	1.0–5.0 (max. 10 μm) 99.9% < 7 μm	2.6 ± 0.2	15	*f* = 4–6 MHz χ = 0.6 N/m	[50,51,52]

EBD = endocardial border definition; LVO = left ventricular opacification; HSA = human serum albumin; DPPC = 1,2-dipalmitoyl-sn-glycero-3-phosphocholine; DPPA = 1,2-dipalmitoyl-sn-glycero-3-phosphate; DPPE-mPEG5000 = 1,2- dipalmitoyl-sn-glycero-3-phosphoethanolamine-N-[methoxy(polyethylene glycol)-5000]; DSPC = 1,2-distearoyl-sn-glycero-3-phosphocholine; DPPG = 1,2-dipalmitoyl-sn-glycero-3-phospho-(1′-rac-glycerol); PA = palmitic acid; H-EPS = hydrogenated egg phosphatidylserine sodium; *ψ*_0_ = zeta potential measured; C_3_F_8_ = perfluoropropane; SF_6_ = sulfur hexafluoride, C_4_F_10_ = perfluorobutane; MBs = microbubbles; **^a^** Bolus intravenous injection; *f* = resonance frequency; χ = shell elasticity. Adapted with permission from references [4,5,21].

At the vascular level, increased transport of therapeutic molecules from the vessel lumen into the surrounding tissue parenchyma also occurs by sonoporation, including the mechanisms described above for uptake into the endothelium and then transcytosis or disruption of the endothelium (e.g., rupture of tight junctions to create transport paths between endothelial cells). This effect can be captured by an increase in the Biot number (*Bi*), which is defined as

(1) $$Bi=\frac{K_{m}L_{tiss}}{D_{tiss}}$$

where $K_{m}$ is the mass transfer coefficient, equivalent to the endothelial cell permeability (m/s), and is proportional to the pharmacokinetic first-order rate constant ($K=K_{m}A$, where A is the endothelial surface area) between the central and tissue compartments; $D_{tiss}$ is the drug diffusivity in the tissue; and $L_{tiss}$ is the diffusion length in tissue, typically the half-length between capillaries (~100 µm). Microbubble sonoporation increases $K_{m}$ in a dose-dependent manner, making diffusion into tissue the limiting transport barrier [53]. Here, the dose includes not only the drug dose, but also the microbubble dose (microbubble volume dose) and ultrasound dose (mechanical index), which can be estimated by the received echo intensity by passive cavitation detection.

Therapeutic agents can be co-administered with MBs or loaded onto the MB shell. This review focuses on shell modification to bioconjugate molecular ligands, drugs (including nucleic acids), nanoparticles and/or cells for therapeutic applications. Articles utilizing so-called “nanobubble” formulations (<1 μm diameter) were excluded from this review. By incorporating specific targeting moieties into the shell, MBs can be engineered to create targeted formulations (tMBs) that exhibit improved pharmacokinetic and acoustic performance, ligand-receptor targeting, biological activation, immunomodulation, and many other functions. With so many options for MB composition, processing, size, microstructure and properties, there is a wealth of opportunity to synthesize innovative targeted MB designs for enhanced performance. Thus, the aim of this review is to summarize the state-of-the-art of current engineered MB formulations and their ultrasound-targeted delivery in different biomedical applications.

## 2. Microbubble Formulations

### 2.1. Shell Composition

#### 2.1.1. Phospholipid-Coated Microbubbles

Phospholipid-coated MBs have a thin and soft shell (3–5 nm) (Figure 1) that provides excellent acoustic response for US-contrast imaging. However, for prolonged imaging and drug delivery, the MBs have limited circulation persistence and drug loading capacity [3,54,55]. To optimize MB performance by controlling their structure, properties, targeting, drug loading, and acoustic response, the shell is made up of saturated diacyl phosphatidylcholine lipids (PC) (80–90 mol%) and PEG-lipid emulsifiers (10–20 mol%) [56]. Longer lipid chains increase intermolecular forces between the phospholipids [57], increase shell rigidity [58,59], decrease shell permeability [60,61], and improve MB stability [62,63,64]. This results in superior acoustic stability, stiffer shell elasticity, and reduced fragmentation propensity [62,65]. They also prolong the in vivo half-life [63] and increase the response at the second harmonic frequency [62,65]. Moreover, MBs with long lipid chains can achieve high delivery efficiency for larger molecules [66].

MBs can be quickly eliminated by opsonization, leading to decreased circulation time. To address this issue, buried-ligand architecture has been developed to extend lipid-coated MB circulation time [67,68,69], preserve targeted specificity, and reduce the immunogenic response by blocking complement protein C3b fixation [67,70,71].

#### 2.1.2. Protein-Coated Microbubbles

Protein-coated MBs have a thicker and stiffer layer (15–150 nm) composed of natural proteins (Figure 1). These MBs exhibit moderate rigidity and drug-loading capabilities, as well as good stability and moderate acoustic response [3,72,73]. At present, only Optison is clinically approved and commercially available as a protein-coated MB for UCAs (Table 1). However, the development of novel albumin-tMBs is gaining interest due to their potential for rapid approval and clinical translation as a drug delivery system.

To maximize albumin-MBs’ loading efficiency, the physicochemical properties of drugs play a critical role. Drug loading can be increased by pre-loading the drugs prior to MB synthesis or using drugs with high albumin binding percentages at higher feed ratios [74]. Additionally, chemical modifications such as disulfide bonds and glutaraldehyde cross-linking [75] can link therapeutic molecules or other loaded carriers to albumin-MBs. Physical properties such as size, shape, storage stability, and acoustic response can be tuned by incorporating dextrose [74,76,77], glycerol, and propylene glycol [77], amphipathic molecules [78], polymers [79], PEGylation [80] or increasing the number of thiols groups [81] into the albumin shell. Several in vitro and in vivo studies have employed these methods to propose using of albumin-MBs as targeted carriers [82,83,84,85,86,87] and multimodal contrast agents [84,86,88,89,90,91]. These strategies offer promising options to develop targeted albumin-MB formulations that can enhance their performance in US-image-guided drug delivery applications. Additionally, proteins such as lysozyme and oleosin [92] are alternative candidates that could be used to create new protein-tMB constructs.

#### 2.1.3. Polymer-Coated Microbubbles

Polymer-coated MBs are composed of a thick and stiff layer (50–500 nm) of biodegradable, natural or synthetic polymers (Figure 1). These MBs can encapsulate either hydrophilic and hydrophobic molecules, carry higher drug doses and enhance MB stability during circulation, more so than typical protein and phospholipid MBs. Nevertheless, their incompressibility and rigidity lead to suboptimal US imaging capability and limited acoustic response [3,72,93]. To enhance polymer-MB drug loading capacity and acoustic properties, their chemical composition or polymer molecular weight (MW) is often modified to adjust the shell’s elasticity and thickness. Increasing the drug’s hydrophobicity and molecular weight also boosts loading capacity, stability, and drug release [94]. Proteins [95] or high MW surfactants [96] can also be used to fine-tune MB loading capacity and acoustic response by regulating their stability, size, and shell thickness.

### 2.2. Gas Composition

The composition of the gas core plays a significant role in the stability of MBs. MBs composed of PFC and SF_6_ gases, which have lower solubility in blood and higher molecular weight, diffuse more slowly across the MB shell, leading to longer circulation persistence. A recent study has shown that MBs filled with C_3_F_8_ or C_4_F_10_ were highly stable, exhibited sustained in vivo circulation, and demonstrated more efficient delivery of Evans blue into the brain without causing side effects as compared to SF_6_-MBs [97].

MBs are now being used as carriers for therapeutic gases, such as NO and O_2_, to potentially regulate the tumor microenvironment or to enhance immune responses. O_2_-MBs, for example, have been used to locally release O_2_ into tumors, thereby improving therapeutic efficacy by reducing hypoxic treatment resistance [98,99]. Additionally, O_2_-MBs can normalize dysfunctional vessels, enhancing vascular maturity, blood perfusion, and drug penetration [100]. Meanwhile, NO-MBs can control the release of NO to mitigate oxidative stress and apoptosis during ischemia–reperfusion injury [101], enhance the targeted delivery of mesenchymal cells into the infarcted myocardium, and induce regional angiogenic response [15]. In addition, NO-MBs accelerate deep vein thrombosis resolution by reducing platelets and inflammatory cells aggregation, enhancing collagen turnover and stimulating an anticoagulant condition of endothelium [102]. 

The use of O_2_- and NO-MBs is still limited due to the properties of the diffusible gases, resulting in lower storage stability and significantly shorter circulation persistence than PFC-MBs. To solve this problem, our group has demonstrated that the lipid shell can be engineered with longer acyl chains (C22:0) to increase O_2_ payload and enhance local delivery [103]. Additionally, O_2_-MBs with larger diameters (2–10 µm) have demonstrated an 8-fold increase in half-life compared to smaller diameters (0.5–2 µm) [104]. Recent approaches propose incorporating folate ligands or SPIONs into drug-loaded-O_2_MBs to accumulate them in a target region by molecular targeting or using an external magnetic field. This incorporation enhances stability, improves drug delivery, and suppresses tumor growth [105,106,107]. Additionally, C4-d-tMBs composed with a gas core combination of PFC/NO have been successfully used to alleviate cardiac allograft rejection by suppressing thrombosis and inflammatory cell infiltrates, while prolonging the survival time two-fold [108].

### 2.3. Size and Microbubble Volume Dose

**Figure 3:**
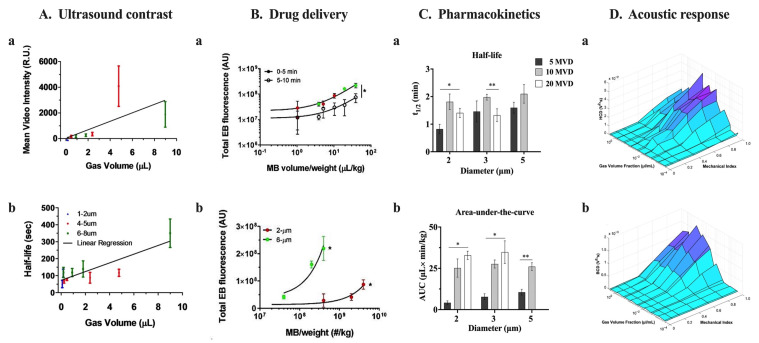
Effect of microbubble volume dose (MVD) on microbubble performance. (**A**) The use of MVD as a dose metric can maximize US imaging contrast (**a**) and circulation persistence (**b**) for isolated microbubbles (MBs) of different diameters. Adapted with permission from reference [112]. Copyright © 2023 World Federation for Ultrasound in Medicine & Biology. Published by Elsevier Inc. All rights reserved. (**B**) The use of MVD results in similar and consistent delivery of Evans blue across the blood–brain barrier (**a**) compared to using MB concentration as dose metric (**b**). The delivery effect of size distribution and concentration of MBs can be collapsed by using MVD. Best fit trend lines, determined by linear regression analysis, are represented by black lines for the 2-µm and 6-µm data sets during the initial 5-minute period of BBB disruption (solid circles; right striatum) and the subsequent 5-minute period (empty circles; left striatum). * *p* < 0.0001. Adapted with permission from reference [116]. (**C**) In vivo pharmacokinetics of size-isolated MBs demonstrated that MVD has greater impact on half-life (**a**) and area-under-the-curve (**b**) than size. * *p* < 0.05 and ** *p* ≤ 0.01. Adapted with permission from [117]. Copyright © 2023, American Chemical Society. (**D**) The in vitro harmonic (**a**) and broadband (**b**) cavitation dose (HCD and BCD) versus mechanical index versus gas volume fraction can be aligned by matching the MVD for 2, 3, and 5 μm diameter and polydisperse MBs. Adapted with permission from [37]. © 2023 by the authors.

The varying components of MBs, including the shell, gas core, size, concentration, and recommended dosage, lead to distinct acoustic responses [109,110], circulation half-life [110,111], and biological effects [111,112,113,114] for both clinically approved MBs (Table 1) and experimental tMB formulations (Table 2, Table 3, Table 4, Table 5 and Table 6). The biological impact of MB acoustic behavior is dose-dependent [115], so selecting dosing strategies for each MB formulation that produce comparable bioeffects is crucial. The MB gas volume dose (MVD) is a unified dose metric that integrates MB size distribution and concentration into a single parameter [22,116]. Our group has demonstrated that MVD can be used to: (1) maximize US imaging contrast and circulation persistence (Figure 3A) [112]; (2) achieve similar, consistent, and comparable molecular delivery across the blood–brain barrier (Figure 3B) [116]; (3) predict, compare, and characterize the MB pharmacokinetics behavior (Figure 3C) [117]; and (4) match the harmonic and broadband cavitation doses regardless of MB size (Figure 3D) [37]. Moreover, matching the gas volume fractions of different commercial MBs with varying sizes and distributions results in equivalent permeabilization effects [118].

The acoustic response of MBs is significantly affected by their size. Changes in size can lead to variations in important parameters such as cavitation threshold [153], radiation force experienced [154,155], and resonance frequency [156,157]. Monodisperse tMB size distributions, combined with acoustic radiation force, have been suggested as a new approach to improve MB targeting, promote ligand–receptor interactions and enhance the sensitivity of US detection in various disease models, including inflammation [158,159,160,161], prostate cancer [162], and fibrosarcoma tumors [67,163]. Our recent results have shown that monodisperse tMBs, driven near their resonance frequency and at low concentrations, can maximize the adhesion efficiency and specificity of tMB [164]. Thus, the use of monodisperse tMB formulations and MVD as a dose metric could be advantageous in future biomedical applications to enhance US-image contrast sensitivity, increase MB adhesion efficiency, maximize MB interaction with endothelial cells, control cavitation behavior, and increase drug delivery efficacy at the target site.

### 2.4. Methods to Control Microbubble Size Distributions

Regulating the size of MBs is critical to control their acoustic response, generate high contrast and maximize therapeutic efficiency. Therefore, ensuring a narrow size distribution is desirable for future formulations. Although mechanical agitation is currently employed to produce clinically approved MB suspensions, the resulting size distributions are highly polydisperse, which is unsuitable for therapeutic applications. Hence, to engineer monodisperse MB suspensions, research has mainly focused on employing differential centrifugation and microfluidic technologies.

#### 2.4.1. Differential Centrifugation

To obtain stable size-selected MBs with narrow size distributions, our group developed the differential centrifugation method [165]. This method involves two steps: First, highly concentrated, polydisperse MB suspensions (10^9^ to 10^10^ MBs/mL) are rapidly produced by the sonication method. Second, the MBs are isolated based on their migration in a centrifugal field. The sorting process relies on the application of Stokes’ equation (Equation (2)) which describes the velocity at which a buoyant particle raises relative to the surrounding fluid under conditions of creeping flow:

(2) $$u_{i}=\frac{2\left(\rho_{2}-\rho_{1i}\right)}{9\eta_{2}}r_{i}^{2}g$$

where *i* refers to the MB size index desired for isolation, *r_i_* is the MB radius, *g* is the relative centrifugal force (RCF) required for the MB rise through a column of length L, and $\eta_{2}$ is the effective viscosity.

Size-isolated MB populations obtained by this method can be biochemically targeted with specific ligands for ultrasound molecular purposes [67,68,69,70,164]. Thus, this method represents a simple way to generate size-isolated tMBs suspensions in high yield that can be adopted for therapeutic applications.

#### 2.4.2. Microfluidic Devices

Microfluidic devices, such as flow focusing [2,166,167,168] and T-junctions [169], have emerged as promising one-step methods to generate stable monodisperse MB suspensions in the clinical application range. In these geometries, a gas phase is concentrated between two liquid flows through an orifice. Due to unstable capillary instability, the gas becomes unstable and pinches off, resulting in the release monodisperse MBs. Moreover, these devices, offer precise control over the flow rate, viscosity, and interfacial tension, allowing for the production of MBs with uniform and narrow size distributions at the desired size [170,171,172,173]. However, one must account for a transient Ostwald ripening process when using microfluidic microbubbles [2,167].

The use of microfluidic devices has been proposed for the in situ production of monodisperse drug-loaded MBs for delivery applications. The use of US and MB enhances the drug delivery to ex vivo rat aortic smooth muscle cells, with over 70% of cells internalization under physiological flow and shear stress conditions [174]. Recently, the feasibility of coating monodisperse MBs with liposomes containing quantum dots and a drug model via a biotin-streptavidin linkage using microfluidic devices has been demonstrated [175]. This technology allows the production of the liposome-MB complex at clinically relevant concentrations with high reproducibility. These results suggest the possibility of adapting this method to simultaneously produce future tMB formulations with homogenous ligand and therapeutic molecule distribution, high payloads, and deliver therapeutic agents in real time.

## 3. Targeted Microbubbles for Therapeutic Applications

**Figure 4:**
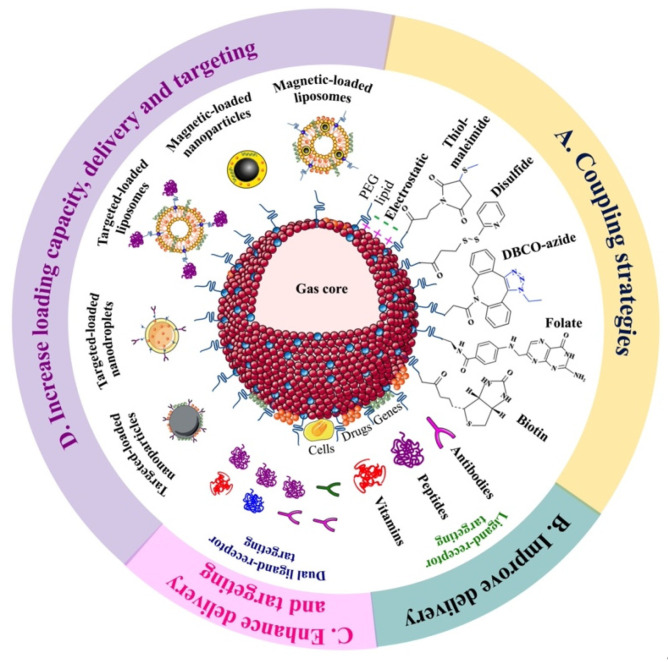
Schematic illustration of current strategies to improve the performance of targeted microbubbles (tMBs) in therapeutic applications. (**A**) Microbubble targeting can be achieved through electrostatic interactions or through the use of PEGylated lipids linked with conjugation groups such as thiol-maleimide, disulfide, DBCO-azide, folate or biotin. (**B**) Ligand–receptor targeting of drug, gene or cell-loaded MBs with antibodies, peptides or vitamins can improve delivery at the target site. (**C**) Dual targeting of loaded MBs with antibody-antibody, antibody-peptide, peptide-peptide, or peptide-vitamin conjugation can improve delivery efficiency and spatial targeting. (**D**) MBs conjugated with molecular or magnetic targeted carriers, such as nanoparticles, liposomes, and nanodroplets, can increase drug loading capacity and delivery efficiency at the target site.

MBs can be biochemically targeted by loading their shell with ligands that avidly bind to specific cell receptors, e.g., those overexpressed during disease states. The ligand can be linked to the MB shell by covalent or noncovalent coupling [6,176,177]. The covalent coupling uses PEG-functionalized lipids with thiol-maleimide, disulfide, DBCO-azide, and folate bonds, whereas noncovalent coupling uses electrostatic interactions and biotin-avidin bridge interactions (Figure 4A).

To improve the delivery of drugs, genes, and cells in different biomedical applications, MB targeting can be divided into four strategies: (1) MBs targeted with a therapeutic ligand; (2) drug or gene loaded-MBs targeted with a ligand; (3) drug, gene, or cell loaded-MBs targeted with two different ligands; and (4) MBs conjugated with magnetically or molecularly targeted loaded carriers.

### 3.1. Microbubbles Targeted with a Therapeutic Ligand

MBs can be loaded with a therapeutic ligand (i.e., where the ligand aids microbubble adhesion *and* has a therapeutic effect) to improve targeted binding efficiency, spatial delivery, and facilitate real-time imaging of tissue structure, function, and molecular characteristics in neurodegenerative and cardiovascular diseases (Table 2). Zhu et al. [20] demonstrated that administering anti-Aβ-tMBs with neural stem cells is a safe and effective dual delivery approach to accelerate Aβ plaque clearance, increase BDNF expression, and improve learning and spatial memory function impairment in Alzheimer’s disease. Similarly, Gao et al. [119] showed that injecting anti-SDF-1-tMBs with mesenchymal stem cells can simultaneously monitor real-time renal perfusion, promote cell homing, and repair early diabetic neuropathy kidneys. Using GPVI-tMBs [120] and anti-IL8-tMBs [121] can aid in diagnosing atherosclerotic lesions, slowing down atheroprogression, reducing inflammation, and enhancing plaque stability. Additionally, administering nitric oxide-filled MBs that are targeted with anti-Cd4 can help alleviate cardiac allograft rejection by suppressing thrombosis and inflammatory cell infiltrates, while prolonging the survival time two-fold [108].

### 3.2. Drug or Gene Loaded-MBs Targeted with a Ligand

Drug- or gene-loaded MB can be targeted with a ligand (here, just to aid microbubble adhesion) to enable site-specific delivery, enhance therapeutic efficacy, and enable image-guided treatment (Figure 4B). Antibody/peptide-tMBs loaded with various genes and chemotherapeutic drugs (Table 3) have been proposed in several studies as successful treatments for various tumors, including glioma [122,123], hind limb [124], and breast tumor [127]. Targeted delivery in these therapies acts as both a molecular contrast agent and a delivery system, improving tumor-specific targeting without harming normal tissues, and increasing local agent delivery, circulation times, and median survival times in mouse and rat models.

A recent study has suggested using folate-tMBs loaded with oxygen and paclitaxel as a dual-targeted system for ovarian tumor cells and tumor-associated macrophages, resulting in two-fold increase in drug concentration, apoptosis index, and median survival [105]. Similarly, folate-tMB loaded with pFLuc enhanced gene transfection efficiency in the brain by 1.5-fold higher than MBs without folate conjugation [129].

On the other hand, different studies have demonstrated that RGD-tMBs loaded with fibrinolytic agents, such as urokinase [125] and tPA [128], can improve arterial blood flow and reduce thrombus size locally without causing adverse side effects, making it a promising and safe option for thrombolysis therapy. Furthermore, VCAM-1-tMBs loaded with mir-126 can serve as a non-invasive and risk-free anti-inflammatory therapy to prevent the growth of abdominal aortic aneurysms and assess the inflammatory state of the endothelium [126].

### 3.3. Drug-, Gene- or Cell-Loaded MBs Targeted with Two Different Ligands

Drug-, gene- or cell-loaded MB can be targeted with two different ligands (dual-tMBs) to achieve higher adhesion efficiency, enhance ultrasound contrast signal, and have more control over drug delivery at the target site (Figure 4C, Table 4). Adipose-derived stem cells conjugated with CD90/ICAM-1-tMBs, known as StemBells, have been proposed as an image-guided treatment for myocardial infarction (MI) [130] and to prevent atherosclerosis acceleration post-MI [131]. Studies have shown that StemBells can improve cardiac function, reach the infarct area without complications, increase cap thickness, decrease intra-plaque macrophage density, and promote the presence of anti-inflammatory macrophages and chemokines in both the plaque and infarcted myocardium, as well as in circulating monocytes.

Additionally, dual-tMBs loaded with genes or chemotherapeutic agents have shown superior performance in treating breast and pancreatic cancer compared to those conjugated with only one ligand [132,133,134]. These studies have shown that dual-tMBs had 2–3 times higher adhesion efficiency, resulting in a 2–4 times greater ultrasound contrast signal for tumor visualization. Additionally, localized release has improved the accumulation of molecules in the tumor region, resulting in tumor suppression 1.5–4 times greater. This has led to reduced side-effects and better drug tolerability.

### 3.4. MBs Conjugated with Magnetically or Molecularly Targeted Loaded Carriers

MBs can be conjugated with other magnetically [106,107,135,136,137,138,139] or molecularly [140,141,142,143] targeted drug/gene-loaded carriers such as nanoparticles, liposomes, and nanodroplets to increase drug loading capacity, stability in circulation, and delivery efficiency (Figure 4D, Table 5). Drug-loaded superparamagnetic iron oxide nanoparticles (SPIONs) conjugated with MBs have been developed to control drug delivery in brain tumors through magnetic targeting and enabling direct delivery visualization using magnetic resonance imaging (MRI) [107,136]. This conjugation provided significant superparamagnetic/acoustic properties for imaging, resulting in a two-fold increase in MB half-life and US contrast signal, as well as 2–4 times more drug and SPION deposition in the tumor region, leading to enhanced MRI signals.

Recently, a magnetically responsive and ultrasound-sensitive delivery system, called doxorubicin-loaded magneto-liposome MBs, has been proposed for enhanced therapy of pancreatic tumors [139]. This combination resulted in a 1.5-fold improvement in drug penetration at the tumor site and a 2-fold increase in tumor suppression compared to the control group without magnetic targeting. Additionally, formulations of drug- or gene- molecularly targeted liposomes conjugated to MBs have shown the potential to enhance the cytotoxic effects in breast, glioma, and colorectal cancer treatment [140,141,142], by increasing the concentration of encapsulated drug in circulation, promoting drug accumulation in tumors, and reducing toxicity in normal tissues. Moreover, liposomes conjugated to MBs, which are molecularly and magnetically responsive, can increase the targeting efficiency to tumor neovasculature, enhance MRI, enhance US tumor imaging by 2.5-fold, prolong the MB half-life by 5-times, and decrease the tumor growth by 2-fold [138].

## 4. Targeted Microbubbles for Immunotherapy Applications

The combination of MBs with US has demonstrated promising potential in modulating and modifying the tumor microenvironment. This is achieved by promoting the penetration of immunotherapeutic agents, enhancing blood perfusion, increasing therapeutic delivery, and inducing tumor cell death [28,30,31,32]. To further enhance the performance of MBs in cancer immunotherapy, the use of loaded-tMBs with drugs, genes or cells has emerged as an attractive strategy. This approach enables precise control of immune stimulation, and it enhances the delivery and pharmacokinetics of immunomodulatory agents at the target site. This strategy has been shown for various cancer immunotherapy modalities, including monoclonal antibodies, immune checkpoint inhibitors, adoptive cell transfer, cytokine therapy, and vaccines (Table 6).

### 4.1. Monoclonal Antibody Immunotherapy

Monoclonal antibody immunotherapy aims to induce cell death by targeting specific antigens, sequences, or epitopes expressed at the disease target site [178]. Therapeutic monoclonal antibodies (mAb) can be administered unconjugated or conjugated with chemotherapeutic drugs and radioisotopes to target tumors and minimize the toxicity effects of conventional chemotherapy [179]. Although mAb immunotherapy has potential therapeutic benefits, poor penetration and heterogenous distribution can impact the therapeutic effectiveness [178,179]. High concentrations are often required, which can result in adverse side effects due to the rapid metabolism and clearance rate through the kidneys [180]. mAb-tMBs + US have been proposed as an image-guided delivery method to increase targeting, enhance local penetration, and potentiate the therapeutic effect of mAbs in different cancer therapies (Table 6). Liao et al. [144] improved glioma treatment by administrating EGFR-tMB + US, resulting in increased tumor vessel permeability and enhanced tumor-suppressing effect 7 days after treatment, with no tumor regrowth in the following 10 days. Kang et al. [145] found that combining anti-DLL4-tMBs + US with DAPT for gastric tumor therapy was more effective than DAPT alone, showing synergistic antitumor proapoptotic effects. These effects were attributed to the regulation of apoptosis-related proteins Bcl-2 and BAX, as well as the tumor suppressor protein P53. Recently, Sun et al. [146] developed a tMB construct that delivers pyropheophorbide sensitizer and therapeutic trastuzumab mAbs for targeted combination of sonodynamic and antibody therapies in gastric cancer. The therapy resulted in enhanced antibody accumulation at the tumor site, increased tumor cell apoptosis and tumor growth inhibition by suppressing AKT phosphorylation.

### 4.2. Immune Checkpoint Inhibitor Therapy

Immune checkpoint inhibitors (ICI) are mAbs variants that activate T cells by blocking immune checkpoint receptors [178]. Approved ICIs for therapy include programmed cell death protein-1 (PD-1), programmed death ligand-1 (PD-L1), and cytotoxic T-lymphocyte-associated antigen-4 (CTLA-4) [178,181]. However, systemic ICI therapy is limited by severe side effects associated with dosage, low treatment response, and overactivation of the immune response [182]. To address these limitations, a controlled delivery strategy called PD-L1 mAb–tMBs + US has been proposed (Table 6). Kim et al. [147] reported that PD-L1 mAb–tMBs can improve therapeutic efficacy, increase the therapeutic index, reduce toxicity, and avoid immune responses and fatalities associated with PD-L1 mAb systemic administration in the treatment of colon cancer. Ma et al. [148] and Liu et al. [149] both demonstrated that combining PD-L1 mAb–tMBs with chemotherapeutic drugs or loading PD-L1 mAb–tMBs with genes exhibited strong synergistic effects in inhibiting cervical tumor growth, improving survival rates, and reducing side effects compared to using either drug/gene or PD-L1 mAb–tMBs alone. The combination treatment showed better immunological activity, indicated by increased CD8+ T cell infiltration and cytokine expression, ultimately resulting in an effective antitumor immune killing effect.

### 4.3. Adoptive Cell Immunotherapy

Adoptive cell-mediated immunotherapy (ACT) involves the intravenous transfer in vitro of resident T cells or genetically modified T cells to target tumor antigens and mediate anti-tumor function. The three types of ACT are tumor-infiltrating lymphocytes (TIL), T cell receptor (TCR) gene therapy, and chimeric antigen receptor-modified T cells (CAR-T) [178,183]. However, ACT is limited by the lack of in vivo persistence of transferred cells, toxicities related to lymphodepletion, immune response, and cytokine release [183]. Preliminary studies using MBs targeted with specific antibodies and retroviruses (CD3, CD8, CD45RA, CD62L, CD3/CD28, and CD-19CAR) have shown that tMBs represent a potential method to stimulate [184,185], activate, transduce, and precisely sort specific phenotypes of CAR-T cells [184]. These stimulations lead to greater in vivo persistence, decreased toxicity, and improved antitumor response of adoptively transferred CAR-T cells when compared to CAR-T cells obtained through conventional methods [185].

### 4.4. Cytokine Immunotherapy

Cytokine-mediated immunotherapy involves the systemic administration of cytokines to enhance the immune response [178]. Commonly used cytokines for immunotherapy in clinics and research include Interferon-alpha (IFN-α), Interleukins (ILs), and granulocyte-macrophage colony-stimulating factor (GM-CSF) [178,186,187]. However, this therapy has several limitations, including low efficacy, high levels of toxicity, and immune response activation [188]. IL-27-tMBs have shown promising results by enhancing cytokine bioactivity, inhibiting prostate tumor growth, and efficiently improving the recruitment of natural killer cells (NKT) and CD8+ cells to the tumor compared to untargeted delivery [189]. Additionally, IL-16–tMBs [190] have been introduced to evaluate myocardial ischemia–reperfusion, detect atherosclerosis, and detect ovarian tumors, respectively.

### 4.5. Vaccine Immunotherapy

Vaccine-mediated immunotherapy involves administering specific antigens or protein fragments to stimulate an immune response [178]. Different types of vaccines are used, including peptide-based vaccines [191], DNA-based vaccines [192], and cell-based vaccines such as NK cells, dendritic cells (DC), and CAR-T cells [193]. However, the effectiveness of vaccine therapy is limited by tissue-specific antigens, low humoral responses, and heterogenous immune responses [178,194]. Gene- and protein-loaded MBs targeted with tumor-specific antigens offer promising image-guided vaccine immunotherapy for breast and melanoma tumors, as demonstrated by Li et al. [150] and Gao et al. [151]. This approach can increase delivery efficiency, prolong survival rates, activate systemic antitumor immunity, inhibit and delay tumor growth [150,151], reduce systemic toxicity, and inhibit cancer metastasis by bridging the innate and adaptive immune responses [150]. Recently, Jungio et al. [152] introduced the first cell-free vaccine in the form of tMBs with activated DC plasma membranes to enhance breast cancer tumor targeting, reduce tumor growth, and increase survival rates. Studies also showed tumor growth inhibition and/or antigen-specific protection through DCs activated by mRNA-loaded MBs [195] or tMBs conjugated with NK cells to promote the controlled delivery at the target site [196].

## 5. Challenges of tMBs in Therapy and Immunotherapy

Table 2, Table 3, Table 4, Table 5 and Table 6 highlight the potential of combining drug-, gene- or cell-loaded tMBs with US for treating various cancers and cardiovascular diseases. However, before exploring future clinical applications, it is crucial to address the in vivo safety of tMBs. The diversity of MB compositions, ligands, therapeutic molecules, and US settings applied pose a significant challenge for clinical translation. While the studies report enhanced therapeutic uptake associated with MB cavitation without side effects, evidence of the latter is lacking. Depending on the US and MB parameters, cavitation can induce adverse bioeffects, such as endothelial cell damage, endothelial dysfunction [197,198], vascular rupture [199,200], and petechial hemorrhages [66,201,202,203]. Moreover, acoustic cavitation can cause long-term side effects on the target tissues [35,197]. Therefore, a deeper understanding of how cavitation events interact with cells and tissues, along with the resulting cellular and molecular responses, can lead to the development of novel design strategies that improve treatment efficacy while minimizing potential safety concerns.

The MB doses and US parameters used in current studies are based on the reference dose of clinically approved MBs, but comparing bioeffects among different MB formulations and animal models is challenging due to the differences in size distribution, dose, shell composition, gas core, and half-life [66,109,111,113,114,204]. Strategies for approximating equivalent MB or cavitation doses are necessary to improve understanding of induced bioeffects. While US and MB doses are critical factors in comparing bioeffects, physiological variations also significantly impact biological outcomes [22]. Therefore, understanding MB pharmacokinetics and accounting for physiological variability is essential for developing safe and consistent treatment protocols.

On the other hand, the reported experimental therapy durations are typically shorter than those in clinic applications and success is limited to reporting tumor growth suppression, tumor cell apoptosis, and survival time. However, MB cavitation can trigger the activation of immune cells, cytokine secretion or protein production, which can modulate and modify the tumor microenvironment to fight against cancer [28,30,31,32]. These immune cells, such as T cells, macrophages, neutrophils, fibroblasts, and B cells, participate in the tumor suppression [205]. Therefore, to explain the molecular mechanisms associated with tumor suppression in detail, it is desirable to extend the analysis to characterize the activation and production of these immune components during therapy and immunotherapy protocols. Moreover, the evaluation of only one or four doses of tMB formulations is insufficient, and further pharmacological and toxicological experiments are necessary to identify the optimal dose, administration frequency and protocols, as well as the US parameters, to increase the delivery and maximize therapeutic effects in future studies.

The majority of experimental studies have used murine-origin monoclonal antibodies linked to the MB surface via avidin and biotin coupling, which could potentially cause severe immune responses in human patients [206,207]. To eliminate immunogenic effects, future studies should consider using humanized antibodies, antibody fragments or peptides linked by covalent binding.

**Figure 5:**
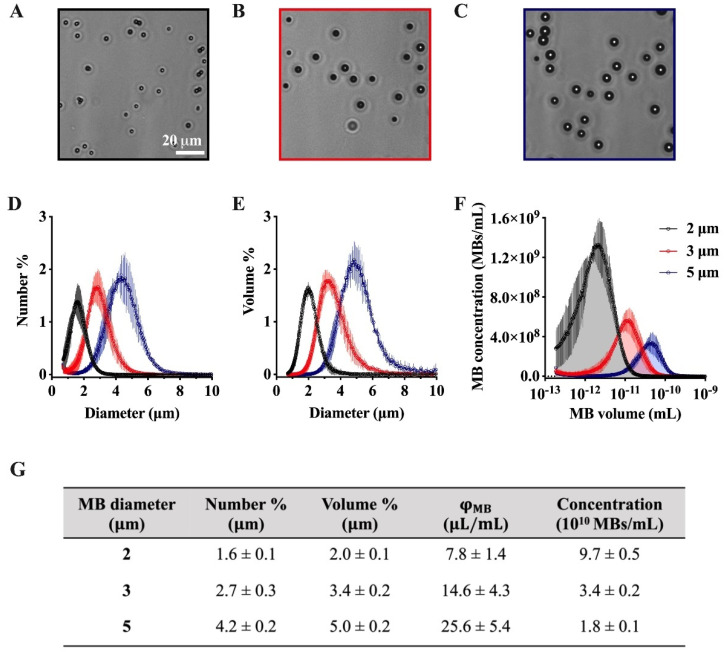
Importance of microbubble volume dose (MVD) as a unifying dose metric. Example of size characteristics for three size-isolated lipid-coated microbubbles: (**A**–**C**) microscope images of MBs from the three different sizes; (**D**) number-weighted size distributions; (**E**) volume-weighted size distributions; (**F**) MB volume vs. concentration curve used to determine the gas volume fraction by taking the area-under-the-curve; (**G**) corresponding size metrics. Adapted with permission from [117]. Copyright © 2023, American Chemical Society.

Finally, the issue of MB dose is oftentimes ambiguous. We have proposed the use of microbubble volume dose (MVD) as a unifying dose metric that combines MB size distribution and concentration dose to provide a useful metric for correlating pharmacokinetic parameters (e.g., half-life), acoustic response, and the amount of drug delivered to target tissue (Figure 3) [116]. For approved, commercially available MB ultrasound contrast agents, the MVD ranges from 6 to 25 µL/kg (Table 1). However, for targeted MBs, the MVD typically ranges from 1 to 50 µL/kg, although some reports have very low MVD (<0.1 µL/kg), while others have very high MVD (>100 µL/kg). Note that these MVDs were calculated by multiplying the dose given to the animal, usually in number of MBs per animal weight (although sometimes animal weights were not reported, so we had to use average for the species and age). Importantly, there is often a significant difference between the number-weighted distribution (Figure 5D) and volume-weighted distribution (Figure 5E). Note the difference in mean diameters, for example (Figure 5G), even for monodisperse size-isolated microbubbles. For example, as a basis of 10^10^ MB/mL, the volume fractions estimated by the number and volume distributions are quite different (208, 350, and 698 µL/kg vs. 406, 699, and 1170 µL/kg for 2, 3, and 5 µm diameter MBs, respectively). Additionally, the size distribution is often skewed from a normal distribution, even for monodisperse MBs, owing to Ostwald ripening and other colloidal mechanisms. Therefore, one should take the area-under-the-curve of the MB volume vs. concentration plot (Figure 5F) to determine the volume fraction ($\emptyset_{MB}$), which can then be used to determine an accurate MVD [117]. Therefore, a major challenge in the field is for researchers to report an accurate MVD for their tMBs, or at least the combination of dose (number of MBs/kg body weight) and volume-weighted mean diameter.

## 6. Future Directions

This review is focused on tMB formulations, which include direct bioconjugation of a ligand, drug, nanoparticle and/or cell onto the MB shell. One may argue that loading onto the MB is more efficient because the payload is administered at a lower dose and is co-localized with the cavitation event. However, it is currently thought that the regulatory burden of approving a specific MB/drug combination for clinical use makes this approach less financially viable. Additionally, MBs are cleared quickly from circulation (typically 5–10 min) with accumulation primarily in the lung, liver, kidney, and spleen, leading to loss and rapid elimination of the therapeutic cargo before it can become available to the target cells [9,34]. Therefore, current clinical trials favor co-administration of commercial MBs (Table 1) and free drug, either as a single cocktail bolus or a sequence of injections. This co-administration without bioconjugation approach is more amenable as a platform, where a single MB formulation could be used for multiple drug/disease indications. However, this simplistic approach also limits the creativity and potential benefits of MB engineering, several examples of which are reviewed here (Table 2, Table 3, Table 4, Table 5 and Table 6). Therefore, it is advantageous to continue to develop novel targeted microbubble formulations and applications.

As discussed in this review, targeted MBs offer many benefits as innovative agents for ultrasound image-guided drug, gene, and cell delivery in therapy and immunotherapy. While research continues on targeted MBs for research purposes in animal models, more work is necessary to improve their safety, efficacy, and financial viability for clinical translation. Further research and advancements should be made in the following areas:

1. The design and characterization of tMB formulations to achieve high payload capacity, stable drug loading, homogenous and reproducible size distributions and colloidal stability, and strong echogenicity and ultrasound responsiveness.
2. The understanding of tMB pharmacokinetics with the goal of extending circulation time and improving biodistribution, and determination of minimum effective dose and maximum tolerated dose for a given drug and US scheme.
3. The evaluation of tMB interactions with cells and tissues, their correlation to short- and long-term bioeffects in vivo, and molecular description of biological mechanisms induced by tMB cavitation.
4. The optimization of US protocols with consideration of tMB pharmacokinetics and bioeffects to ensure treatment safety and efficacy.
5. The establishment of drug, microbubble, and ultrasound dose metrics to compare therapeutic index between tMB formulations.

Innovations in these areas may help reduce over-reliance on commercially available ultrasound contrast agents and promote clinical translation of engineered tMBs.

However, to justify investment into the clinical translation of tMBs, researchers must show a significant improvement in safety and/or efficacy in comparison to currently approved and commercially available MB formulations (see Table 1). For example, it would be helpful to show an increase in therapeutic index for delivery of the target payload using tMBs vs. commercial MBs (vs. systemic administration without MBs). Additionally, it would be helpful for comparison to report dose optimization in terms of microbubble MVD and pharmacokinetics, along with ultrasound MI, duty cycle, and sonication time. Such characterization and reporting would help the community standardize treatments and optimize passive cavitation monitoring methods.

## 7. Conclusions

This review highlights recent developments in targeted MB formulations engineered for drug and cell delivery for therapy and immunotherapy. Basic MB formulations are described prior to citing some recent innovative designs for targeted MBs and their applications. The bioconjugation approach has demonstrated promise in enhancing the delivery of various drugs, genes, and cells to target tissues. Issues for future research are discussed, including the need to better define tMB pharmacokinetics and bioeffects, as well as the standardization of MB and US parameters to compare therapeutic index between different MB formulations.

## Figures and Tables

**Table 2:** Microbubbles targeted with a therapeutic ligand used for therapy.

Ligand Type	Ligand Species	Conjugation Chemistry	Microbubble Composition	Ultrasound Parameters	Animal/Disease Model	Reference
**Protein**	SDF-1α	EDC/sulfo-NHS	Shell: DSPC, DPPG, PEG4000-COOH Gas: C_3_F_8_ Diameter: 1–5 μm Dose: unknown	f = 4 MHz MI = 1.5 Time = 1.15 min	Rat/Diabetic nephropathy	[119]
	GPVI-Fc	Avidin-biotin	MicroMarker™ Shell: Phospholipid Gas: C_4_F_10_/N_2_ Diameter: 2.3–2.9 μm Dose: 2.8 × 10^8^ MBs/kg or 5.3 µL/kg MVD	f = 24 MHz Time = 2 min	Mouse/ Atherosclerosis	[120]
**Antibody**	Aβ 1-42	Avidin-biotin	Shell: DSPC, DPPA, PEG4000 Gas: C_3_F_8_ Diameter: 2.13 μm Dose: 5.8 × 10^8^ MBs/kg or 2.9 µL/kg MVD	f = 3 MHz MI = 0.8 PRF = 50 Hz Time = 5 min	Mouse/Alzheimer’s disease	[20]
	C4d mAb	Avidin-biotin	Shell: DPPC, DSPE-PEG-2000, -Biotin Gas: C_3_F_8_/NO Diameter: 0.96 ± 0.07 μm Dose: 5.8 × 10^6^ MBs/kg or 0.003 µL/kg MVD	f = 13 MHz MI = 0.33 Time = 5 min	Rat/Heterotopic Heart transplant	[108]
	IL-8 mAb	Avidin-biotin	USphere™ Labeler Shell: Phospholipid Diameter: 1.8 μm Dose: 1.2 × 10^9^ MBs/kg or 3.6 µL/kg MVD	f = 1 MHz MI = 1.5 Power = 5 W Time = 1 min	Rabbit/ Atherosclerosis	[121]

DSPC = 1,2-distearoyl-sn-glycero-3-phosphocholine; DPPG = 1,2-dipalmitoyl-sn-glycero-3-phospho-(1′-rac-glycerol); DPPA = 1,2-dipalmitoyl-sn-glycero-3-phosphate; PEG4000-COOH = polyethylene glycol-4000-carboxy; DSPE-PEG2000 = 1,2-distearoyl-sn-glycero-3-phosphoethanolamine-N-[methoxy(polyethylene glycol)-2000]; C_3_F_8_ = perfluoropropane; C_4_F_10_ = perfluorobutane; NO = nitric oxide; MVD = Microbubble Volume Dose; f = frequency; MI = mechanical index; PRF = pulse repetition frequency.

**Table 3:** Drug or gene loaded-MBs targeted with a ligand used for therapy.

Ligand Type	Targeting Ligand	Therapeutic Molecule	Conjugation Chemistry	Microbubble Composition	Ultrasound Parameters	Animal/Disease Model	Reference
**Antibody**	VEGFR2 mAb	pHSV-TK	Avidin-biotin Electrostatic	Shell: DPPC, DPTAP, DSPE-PEG2000, -Biotin Gas: C_3_F_8_ Diameter: 1.1 ± 0.1 μm Dose: 1.8 × 10^10^ MBs/kg or 12.4 µL/kg MVD	f = 1 MHz PNP = 0.7 MPa PRF = 5 Hz Time = 2 min	Mouse/Glioma tumor	[122]
	VEGFR2 mAb	BCNU	Avidin-biotin Hydrophobic	Shell: DPPC, DSPE-PEG2000, -Biotin Gas: C_3_F_8_ Diameter: 1.79 ± 0.13 μm Dose: 1.4 × 10^10^ MBs/kg or 41 µL/kg MVD	f = 1 MHz PNP = 0.7 MPa DC = 5% PRF = 5 Hz Time = 1 min	Rat/Glioma tumor	[123]
	CD105	pEZ-M46-ES	Avidin-biotin Electrostatic	Shell: DPPC, Cholesterol, DSPE-PEG2000, -Biotin Gas: C_3_F_8_ Diameter: 1.44 ± 0.21 μm Dose: 1.3 × 10^10^ MBs/kg or 20.6 µL/kg MVD	f = 1 MHz PNP = 0.7 MPa DC = 50% I = 2 W/cm^2^ PRF = 5 Hz Time = 30 s	Mouse/Hind limb tumor	[124]
	RGD	Urokinase	Electrostatic	SonoVue Diameter: 1.5–2.5 μm Dose: 4.4 × 10^7^ MBs/kg or 0.18 µL/kg MVD	f = 1.6 MHz MI = 1.1 PRF = 24 kHz Time = 10 min	Pig/Thrombosis	[125]
	VCAM-1	miR-126	Avidin-biotin	VisualSonics Target-Ready MBs Shell: Phospholipid Gas: C_4_F_10_/N_2_ Diameter: 1.5 μm Dose: 3.7 × 10^9^ MBs/kg or 6.6 µL/kg MVD	f = 10 MHz MI = 0.66 I = 0.076 W/cm^2^ Time = 2.5 min	Mouse/Aortic Aneurysm	[126]
**Peptides**	LHRa	Paclitaxel	Avidin-biotin Hydrophobic	Shell: DPPC, DSPE-PEG2000-Biotin Gas: C_3_F_8_ Diameter: 1.8 ± 0.2 μm Dose: 1.5 × 10^9^ MBs/kg or 4.5 µL/kg MVD	f = 0.3 MHz I = 1 W/cm^2^ DC = 50% Time = 3 min	Mouse/Breast cancer	[127]
	RGD	tPA	Amine Hydrophobic	Shell: DPPC, DSPC PEG-Amine Gas: C_3_F_8_ Diameter: 2.08 ± 0.93 μm Dose: 8.0 × 10^8^ MBs/kg or 3.8 µL/kg MVD	f = 2 MHz MI = 1.4 I = 1.8 W/cm^2^ DC = 95% PRF = 15 Hz Time = 30 min	Rabbit/ Thrombolysis	[128]
**Vitamins**	Folate	Paclitaxel	Hydrophobic	Shell: DPPC, DSPE-PEG2000-Folate Gas: O_2_ Diameter: 1.81 ± 0.04 μm Dose: unknown	f = 0.3 MHz I = 1 W/cm^2^ Time = 3 min	Mouse/Ovarian cancer	[105]
	Folate	p-FLuc	Amide Electrostatic	Shell: DPPC, DPTAP, DSPE-PEG2000 Gas: C_3_F_8_ Diameter: 3.2 ± 0.1 μm Dose: 1.8 × 10^8^ MBs/kg or 3.1 µL/kg MVD	f = 1 MHz PRF = 5 Hz PNP = 0.7 MPa Time = 1 min	Rat/ Glioma tumor	[129]

DPPC = 1,2-dipalmitoyl-sn-glycero-3-phosphocholine; DSPE-PEG2000 = 1,2-distearoyl-sn-glycero-3-phosphoethanolamine-N-[methoxy(polyethylene glycol) -2000]; DPPG = 1,2-dipalmitoyl-sn-glycero-3-phospho-(1′-rac-glycerol); PA = palmitic acid; DSPC = 1,2-distearoyl-sn-glycero-3-phosphocholine; DPTAP = 1,2-dipalmitoyl-3-trimethylammonium-propane; C_3_F_8_ = perfluoropropane; SF_6_ = sulfur hexafluoride, C_4_F_10_ = perfluorobutane; N_2_ = nitrogen; O_2_ = oxygen; MVD = Microbubble Volume Dose; f = frequency; MI = mechanical index; PRF = pulse repetition frequency; I = intensity; DC = duty cycle; PNP = peak negative pressure.

**Table 4:** Drug, gene, or cell loaded-MBs targeted with two different ligands.

Targeting Ligands	Therapeutic Molecule	Conjugation Chemistry	Microbubble Composition	Ultrasound Parameters	Animal/Disease Model	Reference
**CD90 Ab** **ICAM-1 Ab**	Adipose-derived stem cells	Avidin-biotin	Shell: DSPC, PEG40S, DSPE-PEG2000, -Biotin Gas: C_4_F_10_ Diameter: 3.5 μm Dose: 9.3 × 10^7^ MBs/kg or 2.1 µL/kg MVD	f = 1 MHz PNP: 0.1 MPa PRF = 1 kHz DC = 50% Time = 10 min	Rat/Myocardial Infarction	[130]
**CD90 Ab** **ICAM-1 Ab**	Adipose-derived stem cells	Avidin-biotin	Shell: DSPC, PEG40S, DSPE-PEG2000, -Biotin Gas: C_4_F_10_ Diameter: 3.5 μm Dose: 4.6 × 10^7^ MBs/kg or 1 µL/kg MVD	f = 1 MHz PNP: 0.1 MPa PRF = 1 kHz DC = 50% Time = 1 min	Mouse/ Atherosclerosis	[131]
**CCR2 Ab** **iRGD peptide**	shAKT2	Avidin-biotin, Electrostatic	Shell: DSPC, Stearic-PEI600, DSPE-PEG2000-iRGD-Biotin Gas: C_4_F_10_ Diameter: 1.32 ± 0.22 μm Dose: 5.3 × 10^9^ MBs/kg or 6.3 µL/kg MVD	f = 1 MHz PNP: 1.2 MPa PRF = 1 kHz DC = 50% Time = 10 min	Mouse/Breast cancer	[132]
**cRGD peptide** **Folate**	Doxorubicin	Avidin-biotin	Shell: DSPC, DSPE-PEG2000, -Biotin Gas: C_3_F_8_ Diameter: 5.8 ± 2.1 μm Dose: 1.1 × 10^10^ MBs/kg or 1.1 mL/kg MVD	f = 10 MHz MI: 0.64 Time = 0.5 min	Mouse/Breast cancer	[133]
**cRGD and cCLT1 peptide**	Paclitaxel	Amide, Hydrophobic	Shell: DSPC, DSPE-PEG2000, -COOH Gas: SF_6_ Diameter: 1.59 ± 0.54 μm Dose: 1.0×10^10^ MBs/kg or 21 µL/kg MVD	f = 1 MHz MI = 1.17 DC = 10% I = 2.5 W/cm^2^ Time = 3 min	Mouse/Pancreatic cancer	[134]

DSPC = 1,2-distearoyl-sn-glycero-3-phosphocholine; PEG-40S = Polyoxyethylene (40) stearate; DSPE-PEG2000 = 1,2-distearoyl-sn-glycero-3-phosphoethanolamine-N-[methoxy(polyethylene glycol)-2000]; PEI = Branched Polyethylenimine; C_3_F_8_ = perfluoropropane; SF_6_ = sulfur hexafluoride, C_4_F_10_ = perfluorobutane; MVD= Microbubble Volume Dose; f = frequency; MI = mechanical index; PRF = pulse repetition frequency; I = intensity; DC = duty cycle; PNP = peak negative pressure.

**Table 5:** Microbubbles conjugated with targeted drug-loaded carriers used for therapy.

Carrier	Type of Targeting	Therapeutic Molecule	Conjugation Chemistry	Microbubble Composition	Ultrasound Parameters	Animal/Disease Model	Reference
**Nanoparticles**	Magnetic	5-fluorouracil Rose Bengal	Avidin-biotin	Shell: DBPC, DSPE-PEG2000, -Biotin Gas: O_2_ Diameter: 1–2 μm Dose: 7.0 × 10^9^ MBs/kg or 12.3 µL/kg MVD	f = 1 MHz PNP = 0.85 MPa DC = 30% I = 3.5 W/cm^2^ PRF = 100 Hz Time = 3.5 min	Mouse/Pancreatic tumor	[106]
	Magnetic	Doxorubicin	Amide	Shell: DSPC, DSPG DSPE-PEG2000, -Biotin Gas: C_3_F_8_ Diameter: 5.4 ± 1.1 μm Dose: 5.7 × 10^8^ MBs/kg or 46.7 µL/kg MVD	f = 1 MHz PNP = 0.3 MPa DC = 30% I = 3.5 W/cm^2^ PRF = 1 Hz Time = 4 min	Rat/Glioma tumor	[107]
	Magnetic	Gemcitabine Rose Bengal	Avidin-biotin	Shell: DBPC, DSPE-PEG2000, -Biotin Gas: O_2_ Diameter: 1.9 ± 0.4 μm Dose: 5.4 × 10^9^ MBs/kg or 19.5 µL/kg MVD	f = 1.17 MHz PNP = 0.7 MPa DC = 30% Time = 3.5 min	Mouse/Pancreatic tumor	[135]
	Magnetic	Doxorubicin	Electrostatic and hydrophobic	Shell: DSPC, DSPG DSPE-PEG2000, -Biotin Gas: C_3_F_8_ Diameter: 1.04 ± 0.01 μm Dose: 9.7 × 10^10^ MBs/kg or 56.8 µL/kg MVD	f = 0.4 MHz PNP = 0.325 MPa Power = 4 W PRF = 1 Hz Time = 1.5 min	Rat/Glioma tumor	[136]
	Magnetic	tPA	Electrostatic	Shell: SDS Gas: Air Diameter: 5.36 ± 1.44 μm Dose: 6.4 × 10^10^ MBs/kg or 0.50 µL/kg MVD	f = 18 MHz DC = 10% Time = 5 min	Mouse/ Thrombolysis	[137]
	Magnetic/ molecular	RGD	Amide	Shell: PVA Gas: Air Diameter: 1.37 μm Dose: 5.4 × 10^8^ MBs/kg or 0.70 µL/kg MVD	f = 30 MHz Time = 10 min	Mouse/Colon cancer	[138]
**Liposomes**	Magnetic	Doxorubicin	Maleimide	Shell: DPPC, DSPE-PEG2000-SPDP Gas: C_3_F_8_ Diameter: 4 μm Dose: 7.0 × 10^9^ MBs/kg or 233 µL/kg MVD	f = 1 MHz DC = 30% I = 2 W/cm^2^ Time = 2 min	Mouse/Pancreatic tumor	[139]
	Molecular: RGD peptide	Paclitaxel	Avidin-biotin	Shell: DSPC, DSPE-PEG2000, -Biotin Gas: C_3_F_8_ Diameter: 1.5 μm Dose: 1.1 × 10^10^ MBs/kg or 17.8 µL/kg MVD	f = 1 MHz DC = 1% PRF = 1 Hz Time = 2 min	Mouse/Breast cancer	[140]
	Molecular: NGR peptide	shBirc5	Avidin-biotin	Shell: DPPC, Cholesterol DSPE-PEG2000-Biotin Gas: C_3_F_8_ Diameter: 2.90 ± 0.38 μm Dose: 2.1 × 10^10^ MBs/kg or 265.6 µL/kg MVD	f = 1 MHz DC = 50% Power = 1.84 W Time = 1–5 min	Rat/Glioma tumor	[141]
	Molecular: VEGFR2 Ab	Irinotecan	Avidin-biotin	MicroMarker™ Shell: Phospholipid Gas: C_4_F_10_/N_2_ Diameter: 1.5 μm Dose: 4.7 × 10^9^ MBs/kg or 8.2 µL/kg MVD	f = 2.2 MHz PNP = 0.26 MPa PRF = 1 Hz Time = 4 min	Mouse/Colorectal cancer	[142]
**Nanodroplets**	Molecular: VEGFR2 Ab	Combretastatin A4	Avidin-biotin	Shell: DPPC, DSPE-PEG2000-Biotin Gas: C_4_F_10_ Diameter: 2.6 ± 1.5 μm Dose: unknown	f = 2.2 MHz PNP = 0.26 MPa PRF = 1 Hz Time = 5 s	Mouse/Colorectal cancer	[143]

DBPC:1,2-dibehenoyl-sn-glycero-3-phosphocholine; DSPG: 1,2-Distearoyl-sn-glycero-3-phospho-rac-glycerol; DSPE-PEG2000: 1,2-distearoyl-sn-glycero-3-phosphoethanolamine-N-[methoxy(polyethylene glycol) -2000]; DSPC: 1,2-distearoyl-sn-glycero-3-phosphocholine; SDS: Sodium Dodecyl Sulfate; PVA = Poly(vinyl alcohol); DPPC = 1,2-dipalmitoyl-sn-glycero-3-phosphocholine; C_3_F_8_ = perfluoropropane; C_4_F_10_ = perfluorobutane; O_2_ = oxygen; MVD = Microbubble Volume Dose; f = frequency; PRF = pulse repetition frequency; I = intensity; DC = duty cycle; PNP = peak negative pressure.

**Table 6:** Targeted microbubbles used for cancer immunotherapy.

**Immunotherapy** **Modality**	**Therapeutic** **Molecule**	**Conjugation Chemistry**	**Microbubble** **Composition**	**Ultrasound** **Parameters**	**Animal/Disease Model**	**Reference**
**Monoclonal** **antibody**	EGFR mAb	Avidin-biotin	Targestar™-SA Shell: Phospholipid Gas: C_4_F_10_ Diameter: 2.5 μm Dose: 3.6 × 10^9^ MBs/kg or 29.7 µL/kg MVD	f = 400 kHz PRF = 1 Hz Power = 5 W Time = 3–4 min	Mouse/Glioma tumor	[144]
	DLL4 mAb	Avidin-biotin	Targestar™-SA Shell: Phospholipids Gas: C_4_F_10_ Diameter: 2 μm Dose: 5.3 × 10^8^ MBs/kg or 2.2 µL/kg MVD	f = 1 MHz DC= 50% I = 2 W/cm^2^ Time = 1.5 min	Mouse/Gastric cancer	[145]
	Trastuzumab mAb	NHS	Shell: DPSC, DSPE-PEG-2000-NHS, Cholesterol and pyropheophorbide Gas: SF_6_ Diameter: 1.654 ± 1.07 μm Dose: unknown	f = 1 MHz DC= 50% I = 2 W/cm^2^ Time = 5 min	Mouse/Gastric cancer	[146]
**Immune checkpoint inhibitors**	PDL-1 mAb	NHS	Shell: DSPC, DSPE-PEG-2000-NHS. Gas: SF_6_ Diameter: 1.06 ± 0.31 μm Dose: 6.9 × 10^10^ MBs/kg or 43.3 µL/kg MVD	f = 1.1 MHz DC = 5% PRF = 100 Hz Time = 0.5 min	Mouse/Colon cancer	[147]
	PDL-1 mAb and Cisplatin	Avidin-biotin Unbounded	Shell: DPSC, DSPE-PEG-2000, -Biotin Gas: C_3_F_8_ Diameter: 1.01 ± 0.14 μm Dose: 1 × 10^8^ MBs/kg or 0.06 µL/kg MVD	f = 1 MHz DC = 50% I = 1W/cm^2^ PRF = 1 kHz Time = 1.5 min	Mouse/Cervical cancer	[148]
	PDL-1 mAb and miR-34a	Avidin-biotin Electrostatic	Shell: DSPC, DSPE-PEG-2000, DSPE-PEG-2000-Biotin, PEI-600 Gas: C_3_F_8_ Diameter: 0.940 ± 0.080 μm Dose: 4 × 10^9^ MBs/kg or 1.7 µL/kg MVD	f = 18 MHz DC = 50% I = 1 W/cm^2^ Time = 1.5 min	Mouse/Cervical cancer	[149]
**Vaccine**	CD11b mAb and CGAMP	Maleimide	Shell: DSPC, DSPE-PEG-2000, DSPE-PEG-5000-Maleimide Gas: C_4_F_10_ Diameter: 2.6 μm Dose: 1.4 × 10^9^ MBs/kg or 13.1 µL/kg MVD	f = 1 MHz DC = 50% I = 4 W/cm^2^ Time = 2 min	Mouse/Breast cancer	[150]
	HSP70-MAGEA1	Electrostatic	Shell: Span 60 and Tween 80 Gas: SF_6_ Diameter: 6 μm Dose: 1.3 × 10^9^ MBs/kg or 144.7 µL/kg MVD	MI = 0.75	Mouse/Melanoma tumor	[151]
	Dendritic cell plasma membrane fragments	Hydrophobic	Shell: DPPC, DPPA, DSPE-PEG5000 Gas: C_3_F_8_ Diameter: 1.21 ± 1.0 μm Dose: 5 × 10^8^ MBs/kg or 0.5 µL/kg MVD	f = 18 MHz MI = 0.75 Time = 5 min	Mouse/Breast cancer	[152]

DSPC = 1,2-distearoyl-sn-glycero-3-phosphocholine; DPPA: 1,2-dipalmitoyl-sn-glycero-3-phosphate, DPPC: (1,2-dipalmitoyl-sn-glycero-3-phosphocholine); DSPE: 1,2-Distearoyl-sn-glycero-3-phosphorylethanolamine; PEI: Branched Polyethylenimine; DSPE-PEG-2000: (1,2-distearoyl-sn-glycero-3-phosphoethanolamine-N-methoxypolyethylene glycol)-2000]; DSPE-PEG-5000: (1,2-distearoyl-sn-glycero-3-phosphoethanolamine-N-methoxypolyethylene glycol)-5000]; C_3_F_8_ = perfluoropropane; SF_6_ = sulfur hexafluoride, C_4_F_10_ = perfluorobutane; MVD = Microbubble Volume Dose; f = frequency; DC = duty cycle; I = intensity; PRF = pulse repetition frequency; MI = mechanical index.

## Data Availability

Not applicable.

## References

[1] Epstein P.S., Plesset M.S. (1950). On the Stability of Gas Bubbles in Liquid-Gas Solutions. J. Chem. Phys..

[2] Segers T., Lohse D., Versluis M., Frinking P. (2017). Universal Equations for the Coalescence Probability and Long-Term Size Stability of Phospholipid-Coated Monodisperse Microbubbles Formed by Flow Focusing. Langmuir.

[3] Sirsi S.R., Borden M.A. (2009). Microbubble Compositions, Properties and Biomedical Applications. Bubble Sci. Eng. Technol..

[4] Borden M.A., Song K.-H. (2018). Reverse Engineering the Ultrasound Contrast Agent. Adv. Colloid Interface Sci..

[5] Frinking P., Segers T., Luan Y., Tranquart F. (2020). Three Decades of Ultrasound Contrast Agents: A Review of the Past, Present and Future Improvements. Ultrasound Med. Biol..

[6] Borden M.A., Dayton P.A., Slagle C., Walmer R.W. (2021). Ultrasound Contrast Agents. Molecular Imaging.

[7] Stride E., Segers T., Lajoinie G., Cherkaoui S., Bettinger T., Versluis M., Borden M. (2020). Microbubble Agents: New Directions. Ultrasound Med. Biol..

[8] Sennoga C.A., Kanbar E., Auboire L., Dujardin P.-A., Fouan D., Escoffre J.-M., Bouakaz A. (2017). Microbubble-Mediated Ultrasound Drug-Delivery and Therapeutic Monitoring. Expert Opin. Drug Deliv..

[9] Lammertink B.H.A., Bos C., Deckers R., Storm G., Moonen C.T.W., Escoffre J.-M. (2015). Sonochemotherapy: From Bench to Bedside. Front. Pharmacol..

[10] He J., Liu Z., Zhu X., Xia H., Gao H., Lu J. (2022). Ultrasonic Microbubble Cavitation Enhanced Tissue Permeability and Drug Diffusion in Solid Tumor Therapy. Pharmaceutics.

[11] Escoffre J.-M., Sekkat N., Oujagir E., Bodard S., Mousset C., Presset A., Chautard R., Ayoub J., Lecomte T., Bouakaz A. (2022). Delivery of Anti-Cancer Drugs Using Microbubble-Assisted Ultrasound in Digestive Oncology: From Preclinical to Clinical Studies. Expert Opin. Drug Deliv..

[12] Negishi Y., Endo-Takahashi Y., Maruyama K. (2016). Gene Delivery Systems by the Combination of Lipid Bubbles and Ultrasound. DDT.

[13] Sirsi S.R., Borden M.A. (2012). Advances in Ultrasound Mediated Gene Therapy Using Microbubble Contrast Agents. Theranostics.

[14] Rychak J.J., Klibanov A.L. (2014). Nucleic Acid Delivery with Microbubbles and Ultrasound. Adv. Drug Deliv. Rev..

[15] Tong J., Ding J., Shen X., Chen L., Bian Y., Ma G., Yao Y., Yang F. (2013). Mesenchymal Stem Cell Transplantation Enhancement in Myocardial Infarction Rat Model under Ultrasound Combined with Nitric Oxide Microbubbles. PLoS ONE.

[16] Toma C., Fisher A., Wang J., Chen X., Grata M., Leeman J., Winston B., Kaya M., Fu H., Lavery L. (2011). Vascular Endoluminal Delivery of Mesenchymal Stem Cells Using Acoustic Radiation Force. Tissue Eng. Part A.

[17] Sun T., Gao F., Li X., Cai Y., Bai M., Li F., Du L. (2018). A Combination of Ultrasound-Targeted Microbubble Destruction with Transplantation of Bone Marrow Mesenchymal Stem Cells Promotes Recovery of Acute Liver Injury. Stem Cell Res.

[18] Zhu T., Huang X., Peng S., Ye Y., Zhao J. (2022). Ultrasound Targeted Microbubble Destruction Promotes the Therapeutic Effect of HUMSC Transplantation on Glaucoma-Caused Optic Nerve Injury in Rabbits. Trans. Vis. Sci. Tech..

[19] Cui H., Zhu Q., Xie Q., Liu Z., Gao Y., He Y., Tan X., Xu Y. (2020). Low Intensity Ultrasound Targeted Microbubble Destruction Assists MSCs Delivery and Improves Neural Function in Brain Ischaemic Rats. J. Drug Target..

[20] Zhu Q., Xu X., Chen B., Liao Y., Guan X., He Y., Cui H., Rong Y., Liu Z., Xu Y. (2022). Ultrasound-targeted Microbubbles Destruction Assists Dual Delivery of Beta-amyloid Antibody and Neural Stem Cells to Restore Neural Function in Transgenic Mice of Alzheimer’s Disease. Med. Phys..

[21] Schoen S., Kilinc M.S., Lee H., Guo Y., Degertekin F.L., Woodworth G.F., Arvanitis C. (2022). Towards Controlled Drug Delivery in Brain Tumors with Microbubble-Enhanced Focused Ultrasound. Adv. Drug Deliv. Rev..

[22] Song K.-H., Harvey B.K., Borden M.A. (2018). State-of-the-Art of Microbubble-Assisted Blood-Brain Barrier Disruption. Theranostics.

[23] Wang J., Li Z., Pan M., Fiaz M., Hao Y., Yan Y., Sun L., Yan F. (2022). Ultrasound-Mediated Blood–Brain Barrier Opening: An Effective Drug Delivery System for Theranostics of Brain Diseases. Adv. Drug Deliv. Rev..

[24] Unger E., Porter T., Lindner J., Grayburn P. (2014). Cardiovascular Drug Delivery with Ultrasound and Microbubbles. Adv. Drug Deliv. Rev..

[25] Qian L., Thapa B., Hong J., Zhang Y., Zhu M., Chu M., Yao J., Xu D. (2018). The Present and Future Role of Ultrasound Targeted Microbubble Destruction in Preclinical Studies of Cardiac Gene Therapy. J. Thorac. Dis..

[26] Chen H.H., Matkar P.N., Afrasiabi K., Kuliszewski M.A., Leong-Poi H. (2016). Prospect of Ultrasound-Mediated Gene Delivery in Cardiovascular Applications. Expert Opin. Biol. Ther..

[27] Wanigasekara J., de Carvalho A.M.A., Cullen P.J., Tiwari B., Curtin J.F. (2021). Converging Technologies: Targeting the Hallmarks of Cancer Using Ultrasound and Microbubbles. Trends Cancer.

[28] Tu J., Zhang H., Yu J., Liufu C., Chen Z. (2018). Ultrasound-Mediated Microbubble Destruction: A New Method in Cancer Immunotherapy. OTT.

[29] Li H., Zhang Y., Shu H., Lv W., Su C., Nie F. (2022). Highlights in Ultrasound-Targeted Microbubble Destruction-Mediated Gene/Drug Delivery Strategy for Treatment of Malignancies. Int. J. Pharm..

[30] Han Y., Sun J., Wei H., Hao J., Liu W., Wang X. (2022). Ultrasound-Targeted Microbubble Destruction: Modulation in the Tumor Microenvironment and Application in Tumor Immunotherapy. Front. Immunol..

[31] Ho Y.-J., Li J.-P., Fan C.-H., Liu H.-L., Yeh C.-K. (2020). Ultrasound in Tumor Immunotherapy: Current Status and Future Developments. J. Control. Release.

[32] Omata D., Munakata L., Maruyama K., Suzuki R. (2022). Ultrasound and Microbubble-Mediated Drug Delivery and Immunotherapy. J. Med. Ultrason..

[33] Yang Y., Li Q., Guo X., Tu J., Zhang D. (2020). Mechanisms Underlying Sonoporation: Interaction between Microbubbles and Cells. Ultrason. Sonochem..

[34] Deprez J., Lajoinie G., Engelen Y., De Smedt S.C., Lentacker I. (2021). Opening Doors with Ultrasound and Microbubbles: Beating Biological Barriers to Promote Drug Delivery. Adv. Drug Deliv. Rev..

[35] Qin P., Han T., Yu A.C.H., Xu L. (2018). Mechanistic Understanding the Bioeffects of Ultrasound-Driven Microbubbles to Enhance Macromolecule Delivery. J. Control. Release.

[36] Kooiman K., Vos H.J., Versluis M., de Jong N. (2014). Acoustic Behavior of Microbubbles and Implications for Drug Delivery. Adv. Drug Deliv. Rev..

[37] Martinez P., Bottenus N., Borden M. (2022). Cavitation Characterization of Size-Isolated Microbubbles in a Vessel Phantom Using Focused Ultrasound. Pharmaceutics.

[38] Wischhusen J., Padilla F. (2019). Ultrasound-Targeted Microbubble Destruction (UTMD) for Localized Drug Delivery into Tumor Tissue. IRBM.

[39] Chatterjee D., Sarkar K. (2003). A Newtonian Rheological Model for the Interface of Microbubble Contrast Agents. Ultrasound Med. Biol..

[40] Ja’afar F., Leow C.H., Garbin V., Sennoga C.A., Tang M.-X., Seddon J.M. (2015). Surface Charge Measurement of SonoVue, Definity and Optison: A Comparison of Laser Doppler Electrophoresis and Micro-Electrophoresis. Ultrasound Med. Biol..

[41] Shi W.T., Forsberg F. (2000). Ultrasonic Characterization of the Nonlinear Properties of Contrast Microbubbles. Ultrasound Med. Biol..

[42] Tan J.-K.Y., Pham B., Zong Y., Perez C., Maris D.O., Hemphill A., Miao C.H., Matula T.J., Mourad P.D., Wei H. (2016). Microbubbles and Ultrasound Increase Intraventricular Polyplex Gene Transfer to the Brain. J. Control. Release.

[43] Fix S.M., Nyankima A.G., McSweeney M.D., Tsuruta J.K., Lai S.K., Dayton P.A. (2018). Accelerated Clearance of Ultrasound Contrast Agents Containing Polyethylene Glycol Is Associated with the Generation of Anti-Polyethylene Glycol Antibodies. Ultrasound Med. Biol..

[44] Tu J., Swalwell J.E., Giraud D., Cui W., Chen W., Matula T.J. (2011). Microbubble Sizing and Shell Characterization Using Flow Cytometry. IEEE Trans. Ultrason. Ferroelect. Freq. Contr..

[45] Helfield B.L., Goertz D.E. (2013). Nonlinear Resonance Behavior and Linear Shell Estimates for Definity^TM^ and MicroMarker^TM^ Assessed with Acoustic Microbubble Spectroscopy. J. Acoust. Soc. Am..

[46] Chatterjee D., Sarkar K., Jain P., Schreppler N.E. (2005). On the Suitability of Broadband Attenuation Measurement for Characterizing Contrast Microbubbles. Ultrasound Med. Biol..

[47] Tu J., Guan J., Qiu Y., Matula T.J. (2009). Estimating the Shell Parameters of SonoVue ^®^ Microbubbles Using Light Scattering. J. Acoust. Soc. Am..

[48] Gorce J.-M., Arditi M., Schneider M. (2000). Influence of Bubble Size Distribution on the Echogenicity of Ultrasound Contrast Agents: A Study of SonoVue?. Investig. Radiol..

[49] Wu S.-K., Chu P.-C., Chai W.-Y., Kang S.-T., Tsai C.-H., Fan C.-H., Yeh C.-K., Liu H.-L. (2017). Characterization of Different Microbubbles in Assisting Focused Ultrasound-Induced Blood-Brain Barrier Opening. Sci. Rep..

[50] Sarkar K., Shi W.T., Chatterjee D., Forsberg F. (2005). Characterization of Ultrasound Contrast Microbubbles Using in Vitro Experiments and Viscous and Viscoelastic Interface Models for Encapsulation. J. Acoust. Soc. Am..

[51] Sontum P.C. (2008). Physicochemical Characteristics of Sonazoid^TM^, A New Contrast Agent for Ultrasound Imaging. Ultrasound Med. Biol..

[52] Landmark K.E., Johansen P.W., Johnson J.A., Johansen B., Uran S., Skotland T. (2008). Pharmacokinetics of Perfluorobutane Following Intravenous Bolus Injection and Continuous Infusion of Sonazoid^TM^ in Healthy Volunteers and in Patients with Reduced Pulmonary Diffusing Capacity. Ultrasound Med. Biol..

[53] Valdez M.A., Fernandez E., Matsunaga T., Erickson R.P., Trouard T.P. (2020). Distribution and Diffusion of Macromolecule Delivery to the Brain via Focused Ultrasound Using Magnetic Resonance and Multispectral Fluorescence Imaging. Ultrasound Med. Biol..

[54] Upadhyay A., Dalvi S.V. (2019). Microbubble Formulations: Synthesis, Stability, Modeling and Biomedical Applications. Ultrasound Med. Biol..

[55] Al-Jawadi S., Thakur S.S. (2020). Ultrasound-Responsive Lipid Microbubbles for Drug Delivery: A Review of Preparation Techniques to Optimise Formulation Size, Stability and Drug Loading. Int. J. Pharm..

[56] Borden M.A. (2016). Lipid-Coated Nanodrops and Microbubbles. Handbook of Ultrasonics and Sonochemistry.

[57] Borden M.A. (2019). Intermolecular Forces Model for Lipid Microbubble Shells. Langmuir.

[58] Lum J.S., Dove J.D., Murray T.W., Borden M.A. (2016). Single Microbubble Measurements of Lipid Monolayer Viscoelastic Properties for Small-Amplitude Oscillations. Langmuir.

[59] Kim D.H., Costello M.J., Duncan P.B., Needham D. (2003). Mechanical Properties and Microstructure of Polycrystalline Phospholipid Monolayer Shells: Novel Solid Microparticles. Langmuir.

[60] Borden M.A., Longo M.L. (2004). Oxygen Permeability of Fully Condensed Lipid Monolayers. J. Phys. Chem. B.

[61] Pu G., Longo M.L., Borden M.A. (2005). Effect of Microstructure on Molecular Oxygen Permeation through Condensed Phospholipid Monolayers. J. Am. Chem. Soc..

[62] Borden M.A., Kruse D.E., Caskey C.F., Shukui Z., Dayton P.A., Ferrara K.W. (2005). Influence of Lipid Shell Physicochemical Properties on Ultrasound-Induced Microbubble Destruction. IEEE Trans. Ultrason. Ferroelect. Freq. Contr..

[63] Garg S., Thomas A.A., Borden M.A. (2013). The Effect of Lipid Monolayer In-Plane Rigidity on in Vivo Microbubble Circulation Persistence. Biomaterials.

[64] Borden M.A., Longo M.L. (2002). Dissolution Behavior of Lipid Monolayer-Coated, Air-Filled Microbubbles: Effect of Lipid Hydrophobic Chain Length. Langmuir.

[65] van Rooij T., Luan Y., Renaud G., van der Steen A.F.W., Versluis M., de Jong N., Kooiman K. (2015). Non-Linear Response and Viscoelastic Properties of Lipid-Coated Microbubbles: DSPC versus DPPC. Ultrasound Med. Biol..

[66] Wu S.-Y., Chen C.C., Tung Y.-S., Olumolade O.O., Konofagou E.E. (2015). Effects of the Microbubble Shell Physicochemical Properties on Ultrasound-Mediated Drug Delivery to the Brain. J. Control. Release.

[67] Borden M.A., Streeter J.E., Sirsi S.R., Dayton P.A. (2013). In Vivo Demonstration of Cancer Molecular Imaging with Ultrasound Radiation Force and Buried-Ligand Microbubbles. Mol. Imaging.

[68] Chen C.C., Sirsi S.R., Homma S., Borden M.A. (2012). Effect of Surface Architecture on In Vivo Ultrasound Contrast Persistence of Targeted Size-Selected Microbubbles. Ultrasound Med. Biol..

[69] Borden M.A., Sarantos M.R., Stieger S.M., Simon S.I., Ferrara K.W., Dayton P.A. (2006). Ultrasound Radiation Force Modulates Ligand Availability on Targeted Contrast Agents. Mol. Imaging.

[70] Borden M.A., Zhang H., Gillies R.J., Dayton P.A., Ferrara K.W. (2008). A Stimulus-Responsive Contrast Agent for Ultrasound Molecular Imaging. Biomaterials.

[71] Chen C.C., Borden M.A. (2011). The Role of Poly(Ethylene Glycol) Brush Architecture in Complement Activation on Targeted Microbubble Surfaces. Biomaterials.

[72] Kiessling F., Fokong S., Bzyl J., Lederle W., Palmowski M., Lammers T. (2014). Recent Advances in Molecular, Multimodal and Theranostic Ultrasound Imaging. Adv. Drug Deliv. Rev..

[73] Rudakovskaya P.G., Barmin R.A., Kuzmin P.S., Fedotkina E.P., Sencha A.N., Gorin D.A. (2022). Microbubbles Stabilized by Protein Shell: From Pioneering Ultrasound Contrast Agents to Advanced Theranostic Systems. Pharmaceutics.

[74] Liu M., Dasgupta A., Qu N., Rama E., Kiessling F., Lammers T. (2021). Strategies to Maximize Anthracycline Drug Loading in Albumin Microbubbles. ACS Biomater. Sci. Eng..

[75] Ji J., Ji J., He X. (2011). Preparation of Ultrasound Microbubbles Crosslinked to Albumin Nanoparticles Packaged with Tissue-Type Plasminogen Activator Gene Plasmid and Method of in Vivo Transfection. JEP.

[76] Borrelli M.J., O’Brien W.D., Bernock L.J., Williams H.R., Hamilton E., Wu J., Oelze M.L., Culp W.C. (2012). Production of Uniformly Sized Serum Albumin and Dextrose Microbubbles. Ultrason. Sonochem..

[77] Chen J.L., Dhanaliwala A.H., Dixon A.J., Klibanov A.L., Hossack J.A. (2014). Synthesis and Characterization of Transiently Stable Albumin-Coated Microbubbles via a Flow-Focusing Microfluidic Device. Ultrasound Med. Biol..

[78] Upadhyay A., Dalvi S.V. (2016). Synthesis, Characterization and Stability of BSA-Encapsulated Microbubbles. RSC Adv..

[79] Lentacker I., De Geest B.G., Vandenbroucke R.E., Peeters L., Demeester J., De Smedt S.C., Sanders N.N. (2006). Ultrasound-Responsive Polymer-Coated Microbubbles That Bind and Protect DNA. Langmuir.

[80] Upadhyay A., Dalvi S.V., Gupta G., Khanna N. (2017). Effect of PEGylation on Performance of Protein Microbubbles and Its Comparison with Lipid Microbubbles. Mater. Sci. Eng. C.

[81] Ma X., Bussonniere A., Liu Q. (2017). A Facile Sonochemical Synthesis of Shell-Stabilized Reactive Microbubbles Using Surface-Thiolated Bovine Serum Albumin with the Traut’s Reagent. Ultrason. Sonochem..

[82] Liu X., Gong P., Song P., Xie F., Miller II A.L., Chen S., Lu L. (2018). Fast Functionalization of Ultrasound Microbubbles Using Strain Promoted Click Chemistry. Biomater. Sci..

[83] Narihira K., Watanabe A., Sheng H., Endo H., Feril L.B., Irie Y., Ogawa K., Moosavi-Nejad S., Kondo S., Kikuta T. (2018). Enhanced Cell Killing and Apoptosis of Oral Squamous Cell Carcinoma Cells with Ultrasound in Combination with Cetuximab Coated Albumin Microbubbles. J. Drug Target..

[84] Wang Y.-H., Liao A.-H., Chen J.-H., Chris Wang C.-R., Li P.-C. (2012). Photoacoustic/Ultrasound Dual-Modality Contrast Agent and Its Application to Thermotherapy. J. Biomed. Opt..

[85] Liou Y.-R., Wang Y.-H., Lee C.-Y., Li P.-C. (2015). Buoyancy-Activated Cell Sorting Using Targeted Biotinylated Albumin Microbubbles. PLoS ONE.

[86] Wang Y.-H., Chen S.-P., Liao A.-H., Yang Y.-C., Lee C.-R., Wu C.-H., Wu P.-C., Liu T.-M., Wang C.-R.C., Li P.-C. (2014). Synergistic Delivery of Gold Nanorods Using Multifunctional Microbubbles for Enhanced Plasmonic Photothermal Therapy. Sci. Rep..

[87] Porter T.R., Xie F., Knapp D., Iversen P., Marky L.A., Tsutsui J.M., Maiti S., Lof J., Radio S.J., Kipshidze N. (2006). Targeted Vascular Delivery of Antisense Molecules Using Intravenous Microbubbles. Cardiovasc. Revascularization Med..

[88] Liao A.-H., Wu S.-Y., Wang H.-E., Weng C.-H., Wu M.-F., Li P.-C. (2013). Evaluation of 18F-Labeled Targeted Perfluorocarbon-Filled Albumin Microbubbles as a Probe for MicroUS and MicroPET in Tumor-Bearing Mice. Ultrasonics.

[89] Barmin R., Rudakovskaya P., Gusliakova O., Sindeeva O., Prikhozhdenko E., Maksimova E., Obukhova E., Chernyshev V., Khlebtsov B., Solovev A. (2021). Air-Filled Bubbles Stabilized by Gold Nanoparticle/Photodynamic Dye Hybrid Structures for Theranostics. Nanomaterials.

[90] Maksimova E.A., Barmin R.A., Rudakovskaya P.G., Sindeeva O.A., Prikhozhdenko E.S., Yashchenok A.M., Khlebtsov B.N., Solovev A.A., Huang G., Mei Y. (2021). Air-Filled Microbubbles Based on Albumin Functionalized with Gold Nanocages and Zinc Phthalocyanine for Multimodal Imaging. Micromachines.

[91] Yoon Y.I., Pang X., Jung S., Zhang G., Kong M., Liu G., Chen X. (2018). Smart Gold Nanoparticle-Stabilized Ultrasound Microbubbles as Cancer Theranostics. J. Mater. Chem. B.

[92] Chen Z., Chattaraj R., Pulsipher K.W., Karmacharya M.B., Hammer D.A., Lee D., Sehgal C.M. (2019). Photoacoustic and Ultrasound Dual-Mode Imaging via Functionalization of Recombinant Protein-Stabilized Microbubbles with Methylene Blue. ACS Appl. Bio Mater..

[93] Xiong X., Zhao F., Shi M., Yang H., Liu Y. (2011). Polymeric Microbubbles for Ultrasonic Molecular Imaging and Targeted Therapeutics. J. Biomater. Sci. Polym. Ed..

[94] Liu M., Dasgupta A., Koczera P., Schipper S., Rommel D., Shi Y., Kiessling F., Lammers T. (2020). Drug Loading in Poly(Butyl Cyanoacrylate)-Based Polymeric Microbubbles. Mol. Pharm..

[95] Estifeeva T.M., Barmin R.A., Rudakovskaya P.G., Nechaeva A.M., Luss A.L., Mezhuev Y.O., Chernyshev V.S., Krivoborodov E.G., Klimenko O.A., Sindeeva O.A. (2022). Hybrid (Bovine Serum Albumin)/Poly (*N*-Vinyl-2-Pyrrolidone-*Co*-Acrylic Acid)-Shelled Microbubbles as Advanced Ultrasound Contrast Agents. ACS Appl. Bio. Mater..

[96] Barmin R.A., Dasgupta A., Bastard C., De Laporte L., Rütten S., Weiler M., Kiessling F., Lammers T., Pallares R.M. (2022). Engineering the Acoustic Response and Drug Loading Capacity of PBCA-Based Polymeric Microbubbles with Surfactants. Mol. Pharm..

[97] Omata D., Maruyama T., Unga J., Hagiwara F., Munakata L., Kageyama S., Shima T., Suzuki Y., Maruyama K., Suzuki R. (2019). Effects of Encapsulated Gas on Stability of Lipid-Based Microbubbles and Ultrasound-Triggered Drug Delivery. J. Control. Release.

[98] Sun J., Yin M., Zhu S., Liu L., Zhu Y., Wang Z., Xu R.X., Chang S. (2016). Ultrasound-Mediated Destruction of Oxygen and Paclitaxel Loaded Lipid Microbubbles for Combination Therapy in Hypoxic Ovarian Cancer Cells. Ultrason. Sonochem..

[99] Eisenbrey J.R., Shraim R., Liu J.-B., Li J., Stanczak M., Oeffinger B., Leeper D.B., Keith S.W., Jablonowski L.J., Forsberg F. (2018). Sensitization of Hypoxic Tumors to Radiation Therapy Using Ultrasound-Sensitive Oxygen Microbubbles. Int. J. Radiat. Oncol. Biol. Phys..

[100] Ho Y.-J., Chu S.-W., Liao E.-C., Fan C.-H., Chan H.-L., Wei K.-C., Yeh C.-K. (2019). Normalization of Tumor Vasculature by Oxygen Microbubbles with Ultrasound. Theranostics.

[101] Liang Z., Chen H., Gong X., Shi B., Lin L., Tao F., Wu Q., Fang M., Li H., Lu C. (2022). Ultrasound-Induced Destruction of Nitric Oxide–Loaded Microbubbles in the Treatment of Thrombus and Ischemia–Reperfusion Injury. Front. Pharmacol..

[102] Wang C., Yang F., Xu Z., Shi D., Chen D., Dai J., Gu N., Jiang Q. (2013). Intravenous Release of NO from Lipidic Microbubbles Accelerates Deep Vein Thrombosis Resolution in a Rat Model. Thromb. Res..

[103] Kwan J.J., Kaya M., Borden M.A., Dayton P.A. (2012). Theranostic Oxygen Delivery Using Ultrasound and Microbubbles. Theranostics.

[104] Reusser T.D., Song K.-H., Ramirez D., Benninger R.K., Papadopoulou V., Borden M.A. (2020). Phospholipid Oxygen Microbubbles for Image-Guided Therapy. Nanotheranostics.

[105] Luo T., Sun J., Zhu S., He J., Hao L., Xiao L., Zhu Y., Wang Q., Pan X., Wang Z. (2017). Ultrasound-Mediated Destruction of Oxygen and Paclitaxel Loaded Dual-Targeting Microbubbles for Intraperitoneal Treatment of Ovarian Cancer Xenografts. Cancer Lett..

[106] Sheng Y., Beguin E., Nesbitt H., Kamila S., Owen J., Barnsley L.C., Callan B., O’Kane C., Nomikou N., Hamoudi R. (2017). Magnetically Responsive Microbubbles as Delivery Vehicles for Targeted Sonodynamic and Antimetabolite Therapy of Pancreatic Cancer. J. Control. Release.

[107] Fan C.-H., Cheng Y.-H., Ting C.-Y., Ho Y.-J., Hsu P.-H., Liu H.-L., Yeh C.-K. (2016). Ultrasound/Magnetic Targeting with SPIO-DOX-Microbubble Complex for Image-Guided Drug Delivery in Brain Tumors. Theranostics.

[108] Liao T., Li Q., Zhang Y., Yang Z., Huang Z., Han F., Chen X., Yin T., Ren J., Sun Q. (2020). Precise Treatment of Acute Antibody-Mediated Cardiac Allograft Rejection in Rats Using C4d-Targeted Microbubbles Loaded with Nitric Oxide. J. Heart Lung Transplant..

[109] Sonne C. (2003). Differences in Definity and Optison Microbubble Destruction Rates at a Similar Mechanical Index with Different Real-Time Perfusion Systems. J. Am. Soc. Echocardiogr..

[110] Hyvelin J.-M., Gaud E., Costa M., Helbert A., Bussat P., Bettinger T., Frinking P. (2017). Characteristics and Echogenicity of Clinical Ultrasound Contrast Agents: An In Vitro and In Vivo Comparison Study: Comparison of Clinical Ultrasound Contrast Agents. J. Ultrasound Med..

[111] McMahon D., Lassus A., Gaud E., Jeannot V., Hynynen K. (2020). Microbubble Formulation Influences Inflammatory Response to Focused Ultrasound Exposure in the Brain. Sci. Rep..

[112] Sirsi S., Feshitan J., Kwan J., Homma S., Borden M. (2010). Effect of Microbubble Size on Fundamental Mode High Frequency Ultrasound Imaging in Mice. Ultrasound Med. Biol..

[113] Choi J.J., Feshitan J.A., Baseri B., Shougang W., Yao-Sheng T., Borden M.A., Konofagou E.E. (2010). Microbubble-Size Dependence of Focused Ultrasound-Induced Blood–Brain Barrier Opening in Mice In Vivo. IEEE Trans. Biomed. Eng..

[114] Samiotaki G., Vlachos F., Tung Y.-S., Konofagou E.E. (2012). A Quantitative Pressure and Microbubble-Size Dependence Study of Focused Ultrasound-Induced Blood-Brain Barrier Opening Reversibility in Vivo Using MRI: FUS-Induced BBB Opening Reversibility. Magn. Reson. Med..

[115] McMahon D., Hynynen K. (2017). Acute Inflammatory Response Following Increased Blood-Brain Barrier Permeability Induced by Focused Ultrasound Is Dependent on Microbubble Dose. Theranostics.

[116] Song K.-H., Fan A.C., Hinkle J.J., Newman J., Borden M.A., Harvey B.K. (2017). Microbubble Gas Volume: A Unifying Dose Parameter in Blood-Brain Barrier Opening by Focused Ultrasound. Theranostics.

[117] Navarro-Becerra J.A., Song K.-H., Martinez P., Borden M.A. (2022). Microbubble Size and Dose Effects on Pharmacokinetics. ACS Biomater. Sci. Eng..

[118] Bing C., Hong Y., Hernandez C., Rich M., Cheng B., Munaweera I., Szczepanski D., Xi Y., Bolding M., Exner A. (2018). Characterization of Different Bubble Formulations for Blood-Brain Barrier Opening Using a Focused Ultrasound System with Acoustic Feedback Control. Sci. Rep..

[119] Gao Y., Wu S., Li L., Wang G., Shen W., Xu Y., Liu Z., Zhuo Z., Xia H., Tan K. (2014). Ultrasound-Targeted Stromal Cell-Derived Factor-1-Loaded Microbubble Destruction Promotes Mesenchymal Stem Cell Homing to Kidneys in Diabetic Nephropathy Rats. IJN.

[120] Metzger K., Vogel S., Chatterjee M., Borst O., Seizer P., Schönberger T., Geisler T., Lang F., Langer H., Rheinlaender J. (2015). High-Frequency Ultrasound-Guided Disruption of Glycoprotein VI-Targeted Microbubbles Targets Atheroprogressison in Mice. Biomaterials.

[121] Yang H., Sun Y., Wei J., Xu L., Tang Y., Yang L., Zhang X., Lu Y. (2019). The Effects of Ultrasound-Targeted Microbubble Destruction (UTMD) Carrying IL-8 Monoclonal Antibody on the Inflammatory Responses and Stability of Atherosclerotic Plaques. Biomed. Pharmacother..

[122] Chang E.-L., Ting C.-Y., Hsu P.-H., Lin Y.-C., Liao E.-C., Huang C.-Y., Chang Y.-C., Chan H.-L., Chiang C.-S., Liu H.-L. (2017). Angiogenesis-Targeting Microbubbles Combined with Ultrasound-Mediated Gene Therapy in Brain Tumors. J. Control. Release.

[123] Fan C.-H., Ting C.-Y., Liu H.-L., Huang C.-Y., Hsieh H.-Y., Yen T.-C., Wei K.-C., Yeh C.-K. (2013). Antiangiogenic-Targeting Drug-Loaded Microbubbles Combined with Focused Ultrasound for Glioma Treatment. Biomaterials.

[124] Zhou Y., Gu H., Xu Y., Li F., Kuang S., Wang Z., Zhou X., Ma H., Li P., Zheng Y. (2015). Targeted Antiangiogenesis Gene Therapy Using Targeted Cationic Microbubbles Conjugated with CD105 Antibody Compared with Untargeted Cationic and Neutral Microbubbles. Theranostics.

[125] Nederhoed J.H., Ebben H.P., Slikkerveer J., Hoksbergen A.W.J., Kamp O., Tangelder G.-J., Wisselink W., Musters R.J.P., Yeung K.K. (2017). Intravenous Targeted Microbubbles Carrying Urokinase versus Urokinase Alone in Acute Peripheral Arterial Thrombosis in a Porcine Model. Ann. Vasc. Surg..

[126] Wang X., Searle A.K., Hohmann J.D., Liu A.L., Abraham M.-K., Palasubramaniam J., Lim B., Yao Y., Wallert M., Yu E. (2018). Dual-Targeted Theranostic Delivery of MiRs Arrests Abdominal Aortic Aneurysm Development. Mol. Ther..

[127] Pu C., Chang S., Sun J., Zhu S., Liu H., Zhu Y., Wang Z., Xu R.X. (2014). Ultrasound-Mediated Destruction of LHRHa-Targeted and Paclitaxel-Loaded Lipid Microbubbles for the Treatment of Intraperitoneal Ovarian Cancer Xenografts. Mol. Pharm..

[128] Hua X., Zhou L., Liu P., He Y., Tan K., Chen Q., Gao Y., Gao Y. (2014). In Vivo Thrombolysis with Targeted Microbubbles Loading Tissue Plasminogen Activator in a Rabbit Femoral Artery Thrombus Model. J. Thromb. Thrombolysis..

[129] Fan C.-H., Chang E.-L., Ting C.-Y., Lin Y.-C., Liao E.-C., Huang C.-Y., Chang Y.-C., Chan H.-L., Wei K.-C., Yeh C.-K. (2016). Folate-Conjugated Gene-Carrying Microbubbles with Focused Ultrasound for Concurrent Blood-Brain Barrier Opening and Local Gene Delivery. Biomaterials.

[130] Woudstra L., Krijnen P.A.J., Bogaards S.J.P., Meinster E., Emmens R.W., Kokhuis T.J.A., Bollen I.A.E., Baltzer H., Baart S.M.T., Parbhudayal R. (2016). Development of a New Therapeutic Technique to Direct Stem Cells to the Infarcted Heart Using Targeted Microbubbles: StemBells. Stem Cell Res..

[131] Woudstra L., Meinster E., Van Haren L., Kay A.M., Koopman M., Belien J.A.M., Morrison M.C., Van Rossum A.C., Helder M.N., Juffermans L.J.M. (2018). StemBell Therapy Stabilizes Atherosclerotic Plaques after Myocardial Infarction. Cytotherapy.

[132] Liu Y., Zhou Y., Xu J., Luo H., Zhu Y., Zeng X., Dong F., Wei Z., Yan F., Zheng H. (2021). Ultrasound Molecular Imaging-Guided Tumor Gene Therapy through Dual-Targeted Cationic Microbubbles. Biomater. Sci..

[133] Luo W., Wen G., Yang L., Tang J., Wang J., Wang J., Zhang S., Zhang L., Ma F., Xiao L. (2017). Dual-Targeted and PH-Sensitive Doxorubicin Prodrug-Microbubble Complex with Ultrasound for Tumor Treatment. Theranostics.

[134] Zhang L., Sun L., Tang Q., Sun S., Zeng L., Ma J., Li X., Ge H., Liang X. (2022). Cascade Drug Delivery through Tumor Barriers of Pancreatic Cancer via Ultrasound in Combination with Functional Microbubbles. ACS Biomater. Sci. Eng..

[135] Beguin E., Gray M.D., Logan K.A., Nesbitt H., Sheng Y., Kamila S., Barnsley L.C., Bau L., McHale A.P., Callan J.F. (2020). Magnetic Microbubble Mediated Chemo-Sonodynamic Therapy Using a Combined Magnetic-Acoustic Device. J. Control. Release.

[136] Fan C.-H., Ting C.-Y., Lin H.-J., Wang C.-H., Liu H.-L., Yen T.-C., Yeh C.-K. (2013). SPIO-Conjugated, Doxorubicin-Loaded Microbubbles for Concurrent MRI and Focused-Ultrasound Enhanced Brain-Tumor Drug Delivery. Biomaterials.

[137] Wang S., Guo X., Xiu W., Liu Y., Ren L., Xiao H., Yang F., Gao Y., Xu C., Wang L. (2020). Accelerating Thrombolysis Using a Precision and Clot-Penetrating Drug Delivery Strategy by Nanoparticle-Shelled Microbubbles. Sci. Adv..

[138] Duan L., Yang F., He W., Song L., Qiu F., Xu N., Xu L., Zhang Y., Hua Z., Gu N. (2016). A Multi-Gradient Targeting Drug Delivery System Based on RGD-l-TRAIL-Labeled Magnetic Microbubbles for Cancer Theranostics. Adv. Funct. Mater..

[139] Dwivedi P., Kiran S., Han S., Dwivedi M., Khatik R., Fan R., Mangrio F.A., Du K., Zhu Z., Yang C. (2020). Magnetic Targeting and Ultrasound Activation of Liposome–Microbubble Conjugate for Enhanced Delivery of Anticancer Therapies. ACS Appl. Mater. Interfaces.

[140] Zhang J., Wang S., Deng Z., Li L., Tan G., Liu X., Zheng H., Yan F. (2018). Ultrasound-Triggered Drug Delivery for Breast Tumor Therapy Through IRGD-Targeted Paclitaxel-Loaded Liposome-Microbubble Complexes. J. Biomed. Nanotechnol..

[141] Zhao G., Huang Q., Wang F., Zhang X., Hu J., Tan Y., Huang N., Wang Z., Wang Z., Cheng Y. (2018). Targeted ShRNA-Loaded Liposome Complex Combined with Focused Ultrasound for Blood Brain Barrier Disruption and Suppressing Glioma Growth. Cancer Lett..

[142] Ingram N., McVeigh L.E., Abou-Saleh R.H., Maynard J., Peyman S.A., McLaughlan J.R., Fairclough M., Marston G., Valleley E.M.A., Jimenez-Macias J.L. (2020). Ultrasound-Triggered Therapeutic Microbubbles Enhance the Efficacy of Cytotoxic Drugs by Increasing Circulation and Tumor Drug Accumulation and Limiting Bioavailability and Toxicity in Normal Tissues. Theranostics.

[143] Charalambous A., Mico V., McVeigh L.E., Marston G., Ingram N., Volpato M., Peyman S.A., McLaughlan J.R., Wierzbicki A., Loadman P.M. (2021). Targeted Microbubbles Carrying Lipid-Oil-Nanodroplets for Ultrasound-Triggered Delivery of the Hydrophobic Drug, Combretastatin A4. Nanomed. Nanotechnol. Biol. Med..

[144] Liao A.-H., Chou H.-Y., Hsieh Y.-L., Hsu S.-C., Wei K.-C., Liu H.-L. (2015). Enhanced Therapeutic Epidermal Growth Factor Receptor (EGFR) Antibody Delivery via Pulsed Ultrasound with Targeting Microbubbles for Glioma Treatment. J. Med. Biol. Eng..

[145] Kang M., Zhang Y., Jin X., Chen G., Huang Y., Wu D., Li G., Shan J., Huang P., Chen J. (2018). Concurrent Treatment with Anti-DLL4 Enhances Antitumor and Proapoptotic Efficacy of a γ-Secretase Inhibitor in Gastric Cancer. Transl. Oncol..

[146] Sun L., Zhang J., Xu M., Zhang L., Tang Q., Chen J., Gong M., Sun S., Ge H., Wang S. (2022). Ultrasound Microbubbles Mediated Sonosensitizer and Antibody Co-Delivery for Highly Efficient Synergistic Therapy on HER2-Positive Gastric Cancer. ACS Appl. Mater. Interfaces.

[147] Kim D., Lee S.S., Moon H., Park S.Y., Lee H.J. (2020). PD-L1 Targeting Immune-Microbubble Complex Enhances Therapeutic Index in Murine Colon Cancer Models. Pharmaceuticals.

[148] Ma Y., Han J., Jiang J., Zheng Z., Tan Y., Liu C., Zhao Y. (2020). Ultrasound Targeting of Microbubble-Bound Anti PD-L1 MAb to Enhance Anti-Tumor Effect of Cisplatin in Cervical Cancer Xenografts Treatment. Life Sci..

[149] Liu Y., Jiang J., Liu C., Zhao W., Ma Y., Zheng Z., Zhou Q., Zhao Y. (2021). Synergistic Anti-Tumor Effect of Anti-PD-L1 Antibody Cationic Microbubbles for Delivery of the MiR-34a Gene Combined with Ultrasound on Cervical Carcinoma. Am. J. Transl. Res..

[150] Li X., Khorsandi S., Wang Y., Santelli J., Huntoon K., Nguyen N., Yang M., Lee D., Lu Y., Gao R. (2022). Cancer Immunotherapy Based on Image-Guided STING Activation by Nucleotide Nanocomplex-Decorated Ultrasound Microbubbles. Nat. Nanotechnol..

[151] Gao X., Nan Y., Yuan Y., Gong X., Sun Y., Zhou H., Zong Y., Zhang L., Yu M. (2018). Gas-filled Ultrasound Microbubbles Enhance the Immunoactivity of the HSP70-MAGEA1 Fusion Protein against MAGEA1-expressing Tumours. Mol. Med. Rep..

[152] Jugniot N., Dahl J.J., Paulmurugan R. (2022). Immunotheranostic Microbubbles (IMBs)—A Modular Platform for Dendritic Cell Vaccine Delivery Applied to Breast Cancer Immunotherapy. J. Exp. Clin. Cancer Res..

[153] Chomas J.E., Dayton P., May D., Ferrara K. (2001). Threshold of Fragmentation for Ultrasonic Contrast Agents. J. Biomed. Opt..

[154] Morgan K.E., Allen J.S., Dayton P.A., Chomas J.E., Klibaov A.L., Ferrara K.W. (2000). Experimental and Theoretical Evaluation of Microbubble Behavior: Effect of Transmitted Phase and Bubble Size. IEEE Trans. Ultrason. Ferroelect. Freq. Contr..

[155] Dayton P.A., Allen J.S., Ferrara K.W. (2002). The Magnitude of Radiation Force on Ultrasound Contrast Agents. J. Acoust. Soc. Am..

[156] Supponen O., Upadhyay A., Lum J., Guidi F., Murray T., Vos H.J., Tortoli P., Borden M. (2020). The Effect of Size Range on Ultrasound-Induced Translations in Microbubble Populations. J. Acoust. Soc. Am..

[157] Segers T., Kruizinga P., Kok M.P., Lajoinie G., de Jong N., Versluis M. (2018). Monodisperse Versus Polydisperse Ultrasound Contrast Agents: Non-Linear Response, Sensitivity, and Deep Tissue Imaging Potential. Ultrasound Med. Biol..

[158] Wang S., Unnikrishnan S., Herbst E.B., Klibanov A.L., Mauldin F.W., Hossack J.A. (2017). Ultrasound Molecular Imaging of Inflammation in Mouse Abdominal Aorta. Invest. Radiol..

[159] Rychak J.J., Klibanov A.L., Ley K.F., Hossack J.A. (2007). Enhanced Targeting of Ultrasound Contrast Agents Using Acoustic Radiation Force. Ultrasound Med. Biol..

[160] Liu J., Zhang P., Liu P., Zhao Y., Gao S., Tan K., Liu Z. (2012). Endothelial Adhesion of Targeted Microbubbles in Both Small and Great Vessels Using Ultrasound Radiation Force. Mol. Imaging.

[161] Wang S., Wang C.Y., Unnikrishnan S., Klibanov A.L., Hossack J.A., Mauldin F.W. (2015). Optical Verification of Microbubble Response to Acoustic Radiation Force in Large Vessels with In Vivo Results. Investig. Radiol..

[162] Frinking P.J.A., Tardy I., Théraulaz M., Arditi M., Powers J., Pochon S., Tranquart F. (2012). Effects of Acoustic Radiation Force on the Binding Efficiency of BR55, a VEGFR2-Specific Ultrasound Contrast Agent. Ultrasound Med. Biol..

[163] Gessner R.C., Streeter J.E., Kothadia R., Feingold S., Dayton P.A. (2012). An In Vivo Validation of the Application of Acoustic Radiation Force to Enhance the Diagnostic Utility of Molecular Imaging Using 3-D Ultrasound. Ultrasound Med. Biol..

[164] Navarro-Becerra J.A., Castillo J.I., Di Ruzza F., Borden M.A. (2023). Monodispersity Increases Adhesion Efficiency and Specificity for Ultrasound-Targeted Microbubbles. ACS Biomater. Sci. Eng..

[165] Feshitan J.A., Chen C.C., Kwan J.J., Borden M.A. (2009). Microbubble Size Isolation by Differential Centrifugation. J. Colloid Interface Sci..

[166] Segers T., Lassus A., Bussat P., Gaud E., Frinking P. (2019). Improved Coalescence Stability of Monodisperse Phospholipid-Coated Microbubbles Formed by Flow-Focusing at Elevated Temperatures. Lab Chip.

[167] Segers T., De Rond L., De Jong N., Borden M., Versluis M. (2016). Stability of Monodisperse Phospholipid-Coated Microbubbles Formed by Flow-Focusing at High Production Rates. Langmuir.

[168] Dhanaliwala A.H., Chen J.L., Wang S., Hossack J.A. (2013). Liquid Flooded Flow-Focusing Microfluidic Device for in Situ Generation of Monodisperse Microbubbles. Microfluid. Nanofluid..

[169] Khan A.H., Jiang X., Kaushik A., Nair H.S., Edirisinghe M., Mercado-Shekhar K.P., Shekhar H., Dalvi S.V. (2022). Combining Ultrasound and Capillary-Embedded T-Junction Microfluidic Devices to Scale Up the Production of Narrow-Sized Microbubbles through Acoustic Fragmentation. Langmuir.

[170] Gañán-Calvo A.M., Gordillo J.M. (2001). Perfectly Monodisperse Microbubbling by Capillary Flow Focusing. Phys. Rev. Lett..

[171] Garstecki P., Stone H.A., Whitesides G.M. (2005). Mechanism for Flow-Rate Controlled Breakup in Confined Geometries: A Route to Monodisperse Emulsions. Phys. Rev. Lett..

[172] Anna S.L., Bontoux N., Stone H.A. (2003). Formation of Dispersions Using “Flow Focusing” in Microchannels. Appl. Phys. Lett..

[173] Wang H., Jiang S., Zhu C., Ma Y., Fu T. (2021). Bubble Formation in T-Junctions within Parallelized Microchannels: Effect of Viscoelasticity. Chem. Eng. J..

[174] Dixon A.J., Dhanaliwala A.H., Chen J.L., Hossack J.A. (2013). Enhanced Intracellular Delivery of a Model Drug Using Microbubbles Produced by a Microfluidic Device. Ultrasound Med. Biol..

[175] Peyman S.A., Abou-Saleh R.H., McLaughlan J.R., Ingram N., Johnson B.R.G., Critchley K., Freear S., Evans J.A., Markham A.F., Coletta P.L. (2012). Expanding 3D Geometry for Enhanced On-Chip Microbubble Production and Single Step Formation of Liposome Modified Microbubbles. Lab Chip.

[176] Klibanov A.L., Schober O., Kiessling F., Debus J. (2020). Ultrasound Molecular Imaging of Cancer: Design and Formulation Strategies of Targeted Contrast Agents. Molecular Imaging in Oncology.

[177] Klibanov A.L. (2005). Ligand-Carrying Gas-Filled Microbubbles: Ultrasound Contrast Agents for Targeted Molecular Imaging. Bioconjugate Chem..

[178] Sambi M., Bagheri L., Szewczuk M.R. (2019). Current Challenges in Cancer Immunotherapy: Multimodal Approaches to Improve Efficacy and Patient Response Rates. J. Oncol..

[179] Cruz E., Kayser V. (2019). Monoclonal Antibody Therapy of Solid Tumors: Clinical Limitations and Novel Strategies to Enhance Treatment Efficacy. BTT.

[180] Hansel T.T., Kropshofer H., Singer T., Mitchell J.A., George A.J.T. (2010). The Safety and Side Effects of Monoclonal Antibodies. Nat. Rev. Drug Discov..

[181] Fares C.M., Van Allen E.M., Drake C.G., Allison J.P., Hu-Lieskovan S. (2019). Mechanisms of Resistance to Immune Checkpoint Blockade: Why Does Checkpoint Inhibitor Immunotherapy Not Work for All Patients?. Am. Soc. Clin. Oncol. Educ. Book.

[182] Singh S., Hassan D., Aldawsari H.M., Molugulu N., Shukla R., Kesharwani P. (2020). Immune Checkpoint Inhibitors: A Promising Anticancer Therapy. Drug Discov. Today.

[183] Rohaan M.W., Wilgenhof S., Haanen J.B.A.G. (2019). Adoptive Cellular Therapies: The Current Landscape. Virchows Arch..

[184] Fu C., Shi G., Liu Y.-T. (2021). Manufacturing Anti-CD19 CAR-Tscm Cells for Immunotherapy Using Innovative Microbubble-Based Technologies for Precision Cell Processing. Blood.

[185] Lustig A., Manor T., Shi G., Li J., Wang Y.-T., An Y., Liu Y.-T., Weng N. (2020). Lipid Microbubble–Conjugated Anti-CD3 and Anti-CD28 Antibodies (Microbubble-Based Human T Cell Activator) Offer Superior Long-Term Expansion of Human Naive T Cells In Vitro. ImmunoHorizons.

[186] Waldmann T.A. (2018). Cytokines in Cancer Immunotherapy. Cold Spring Harb. Perspect. Biol..

[187] Berraondo P., Sanmamed M.F., Ochoa M.C., Etxeberria I., Aznar M.A., Pérez-Gracia J.L., Rodríguez-Ruiz M.E., Ponz-Sarvise M., Castañón E., Melero I. (2019). Cytokines in Clinical Cancer Immunotherapy. Br. J. Cancer.

[188] Qiu Y., Su M., Liu L., Tang Y., Pan Y., Sun J. (2021). Clinical Application of Cytokines in Cancer Immunotherapy. DDDT.

[189] Figueiredo M.L., Figueiredo Neto M., Salameh J.W., Decker R.E., Letteri R., Chan-Seng D., Emrick T. (2020). Ligand-Mediated Targeting of Cytokine Interleukin-27 Enhances Its Bioactivity In Vivo. Mol. Ther.-Methods Clin. Dev..

[190] Barua A., Yellapa A., Bahr J.M., Adur M.K., Utterback C.W., Bitterman P., Basu S., Sharma S., Abramowicz J.S. (2015). Interleukin 16- (IL-16-) Targeted Ultrasound Imaging Agent Improves Detection of Ovarian Tumors in Laying Hens, a Preclinical Model of Spontaneous Ovarian Cancer. BioMed Res. Int..

[191] Malonis R.J., Lai J.R., Vergnolle O. (2020). Peptide-Based Vaccines: Current Progress and Future Challenges. Chem. Rev..

[192] Amara S., Tiriveedhi V. (2017). The Five Immune Forces Impacting DNA-Based Cancer Immunotherapeutic Strategy. Int. J. Mol. Sci..

[193] Saxena M., Balan S., Roudko V., Bhardwaj N. (2018). Towards Superior Dendritic-Cell Vaccines for Cancer Therapy. Nat. Biomed. Eng..

[194] Lin M.J., Svensson-Arvelund J., Lubitz G.S., Marabelle A., Melero I., Brown B.D., Brody J.D. (2022). Cancer Vaccines: The next Immunotherapy Frontier. Nat. Cancer.

[195] Dewitte H., Van Lint S., Heirman C., Thielemans K., De Smedt S.C., Breckpot K., Lentacker I. (2014). The Potential of Antigen and TriMix Sonoporation Using MRNA-Loaded Microbubbles for Ultrasound-Triggered Cancer Immunotherapy. J. Control. Release.

[196] Song H.-W., Lee H.-S., Kim S.-J., Kim H.Y., Choi Y.H., Kang B., Kim C.-S., Park J.-O., Choi E. (2021). Sonazoid-Conjugated Natural Killer Cells for Tumor Therapy and Real-Time Visualization by Ultrasound Imaging. Pharmaceutics.

[197] Navarro-Becerra J.A., Franco-Urquijo C.A., Ríos A., Escalante B. (2021). Localized Delivery of Caveolin-1 Peptide Assisted by Ultrasound-Mediated Microbubble Destruction Potentiates the Inhibition of Nitric Oxide-Dependent Vasodilation Response. Ultrasound Med. Biol..

[198] Navarro-Becerra J.A., Caballero-Robledo G.A., Franco-Urquijo C.A., Ríos A., Escalante B. (2020). Functional Activity and Endothelial-Lining Integrity of Ex Vivo Arteries Exposed to Ultrasound-Mediated Microbubble Destruction. Ultrasound Med. Biol..

[199] Chen H., Brayman A.A., Evan A.P., Matula T.J. (2012). Preliminary Observations on the Spatial Correlation Between Short-Burst Microbubble Oscillations and Vascular Bioeffects. Ultrasound Med. Biol..

[200] Chen H., Brayman A.A., Bailey M.R., Matula T.J. (2010). Blood Vessel Rupture by Cavitation. Urol. Res..

[201] Li P., Armstrong W.F., Miller D.L. (2004). Impact of Myocardial Contrast Echocardiography on Vascular Permeability: Comparison of Three Different Contrast Agents. Ultrasound Med. Biol..

[202] Miller D.L., Lu X., Fabiilli M., Fields K., Dou C. (2016). Frequency Dependence of Petechial Hemorrhage and Cardiomyocyte Injury Induced during Myocardial Contrast Echocardiography. Ultrasound Med. Biol..

[203] White P.J., Zhang Y.-Z., Power C., Vykhodtseva N., McDannold N. (2020). Observed Effects of Whole-Brain Radiation Therapy on Focused Ultrasound Blood–Brain Barrier Disruption. Ultrasound Med. Biol..

[204] Sun T., Samiotaki G., Wang S., Acosta C., Chen C.C., Konofagou E.E. (2015). Acoustic Cavitation-Based Monitoring of the Reversibility and Permeability of Ultrasound-Induced Blood-Brain Barrier Opening. Phys. Med. Biol..

[205] Anderson N.M., Simon M.C. (2020). The Tumor Microenvironment. Curr. Biol..

[206] Hernot S., Klibanov A.L. (2008). Microbubbles in Ultrasound-Triggered Drug and Gene Delivery. Adv. Drug Deliv. Rev..

[207] Jallinoja V.I.J., Houghton J.L. (2021). Current Landscape in Clinical Pretargeted Radioimmunoimaging and Therapy. J. Nucl. Med..

